# A Mitochondria‐Targeted Biomimetic Nanomedicine Capable of Reversing Drug Resistance in Colorectal Cancer Through Mitochondrial Dysfunction

**DOI:** 10.1002/advs.202410630

**Published:** 2025-02-12

**Authors:** Xiaohui Wang, Zhiyuan Xu, Jian Wang, Chunrong Wu, Lin Zhang, Chengyuan Qian, Yang Luo, Yanjuan Gu, Wing‐Tak Wong, Debing Xiang

**Affiliations:** ^1^ Department of Oncology Chongqing University Jiangjin Hospital Chongqing 402260 China; ^2^ Department of Applied Biology and Chemical Technology The Hong Kong Polytechnic University Hong Kong SAR China; ^3^ Department of Electrical and Electronic Engineering The Hong Kong Polytechnic University Hong Kong SAR China; ^4^ Department of Gastroenterology Chongqing University Jiangjin Hospital Chongqing 402260 China; ^5^ Department of Laboratory Medicine, Chongqing General Hospital School of Medicine Chongqing University Chongqing 401147 China

**Keywords:** colorectal cancer, drug resistance, exosome, metastasis, mitochondrial dysfunction

## Abstract

Chemoresistance and metastasis are the main obstacles to the clinical success of anticancer treatment and are responsible for most cancer deaths. Developing effective approaches to reverse chemoresistance and inhibit metastasis is essential for efficient chemotherapy. Mitochondria are important sources of cellular energy and are involved in mediating chemoresistance and driving tumor metastasis. Due to the relatively weak DNA repair capacity of mitochondria, targeting mitochondria may reverse chemoresistance and provide a paradigm for metastatic cancer treatment. Herein, exosomes (Exos) modified with integrin ligands and mitochondriotropic molecules are synthesized for encapsulating oxaliplatin (OXA) to construct a sequentially targeted and mitochondrion‐dysfunctional nanodrug (OXA@Exo‐RD). Subsequent investigations confirm that OXA@Exo‐RD targeted cancer cells and mitochondria in sequence, and OXA delivered to mitochondria lacking DNA repair mechanisms reduce the likelihood of deactivation. Furthermore, the OXA@Exo‐RD promotes the overproduction of ROS, inhibited ATP generation, and induces mitochondria‐mediated apoptosis and mitochondrial dysregulation. Finally, OXA@Exo‐RD shows the potential to inhibit the growth and metastasis of HCT116/OXA cells in vitro, which is further validated in subcutaneous and orthotopic CRC models, as well as in CRC metastasis models. Taken together, this dual‐targeting nanomedicine induces apoptosis via mitochondrial signaling pathways, providing an attractive strategy for the treatment of drug‐resistant CRC.

## Graphical abstract

Chemoresistance and metastasis are the main obstacles to the clinical success of anticancer treatment and are responsible for most cancer deaths in CRC. This study develops a sequentially targeted and mitochondrion‐dysfunctional nanodrug (OXA@Exo‐RD) that simultaneously enhances drug concentration and potency in the targeted organelle to overcome chemoresistance and reduce metastasis, which provides new opportunities for the clinical treatment of CRC.

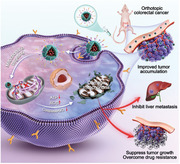

## 1 Introduction

Colorectal cancer (CRC) is one of the most prevalent types of malignant tumors, with increasing morbidity and mortality, which seriously threatens human health and survival.^[^ ^1^, ^2^ ^]^ Oxaliplatin (OXA) is a first‐line chemotherapeutic agent for advanced CRC patients. It targets and binds to DNA to prevent the unwinding of the DNA double helix, thereby inhibiting DNA replication and exerting an anti‐tumor effect.^[^ ^3^ ^]^ Although OXA has good application prospects, its drug resistance and adverse reactions caused by nonspecific distribution limit the therapeutic effect.^[^ ^4^ ^]^ More importantly, metastasis is another obstacle to the effective treatment of CRC, which can cause treatment failure.^[^ ^5^, ^6^, ^7^, ^8^ ^]^

The DNA damage repair mechanism reduces the effectiveness of chemotherapy by repairing the intrachain cross‐links between platinum and DNA, which is one of the main reasons for chemoresistance.^[^ ^9^, ^10^ ^]^ Due to the overexpression of genes pertinent to DNA damage repair in drug‐resistant tumor cells, valid strategies are urgently needed to prevent DNA repair initiation and regain the susceptibilities of drug‐resistant tumor cells to platinum.^[^ ^11^, ^12^ ^]^ To date, several inhibitors related to DNA repair, such as DNA‐PK inhibitors, PARP inhibitors, and MGMT inhibitors, have been applied in combination with platinum in an effort to improve anti‐tumor efficacy.^[^ ^13^, ^14^, ^15^ ^]^ In addition, the study showed that mitochondria have no DNA damage repair function, suggesting that delivering platinum to mitochondria rather than cell nuclei could be a preferred option for chemotherapy.^[^ ^16^, ^17^ ^]^ Therefore, interfering mtDNA in mitochondria may provide a better way to improve treatment efficacy and circumvent drug resistance.^[^ ^10^ ^]^ Increasing evidence suggests that mitochondria are also involved in the occurrence and progression of cancer, and play an important role in cancer cell metastasis. Therefore, targeting mitochondria to induce dysfunction may be a good approach to circumvent chemoresistance and inhibit metastasis.^[^ ^18^, ^19^, ^20^ ^]^

Delocalized lipophilic cations (DLCs) are often used for targeting mitochondria.^[^ ^21^, ^22^ ^]^ The lipophilicity of DLCs enables them to cross the mitochondrial membrane, while their positive charge facilitates them mitochondrial matrix accumulation driven by the transmembrane electric potential, giving them a mitochondrial‐targeting ability.^[^ ^23^, ^24^ ^]^ What's more, since the transmembrane potential of mitochondria in cancer cells is higher than that in normal cells, they can preferentially accumulate in cancer cell mitochondria.^[^ ^25^, ^26^ ^]^ To date, mitochondrial targeting ligands including triphenylphosphonium cation (TPP^+^), dequalinium (DQA), rhodamine 123, and mitochondrial targeting signal peptides (MTSs) have been used in mitochondrial drug delivery systems.^[^ ^27^, ^28^, ^29^ ^]^ As a DLCs molecule, DQA selectively accumulates in cancer cell mitochondria and dissipates mitochondrial membrane potential (MMP), induces reactive oxygen species (ROS) generation and inhibits ATP synthesis, thereby killing cancer cells.^[^ ^30^, ^31^, ^32^ ^]^ Conjugation of therapeutic agents with mitochondrial targeting ligands to achieve precise mitochondrial delivery is a promising strategy. However, these conjugates generally suffer from poor water solubility and are easily eliminated from the body after systemic administration, leading to unideal pharmacokinetic properties.^[^ ^25^ ^]^ What is more, some therapeutic agents may affect their chemical activity after conjugation to mitochondrial targeting ligands.^[^ ^33^, ^34^ ^]^ Hence, it is urgent to find suitable drug carriers to maximize drug activity while precisely targeting mitochondria.

Exosomes (Exos) are naturally derived nanocarriers with the benefits of good biocompatibility, high stability, long circulation, and low immunogenicity. Their phospholipid bilayer structure and unique cargo protection ability make them excellent drug carriers.^[^ ^35^, ^36^, ^37^, ^38^ ^]^ Compared with other cell types, mesenchymal stem cells (MSCs) are ideal candidates for the large‐scale production of exosomes for drug delivery and the creation of exosome‐based chemotherapy platforms for cancer treatment.^[^ ^39^, ^40^, ^41^ ^]^

**Scheme 1:**
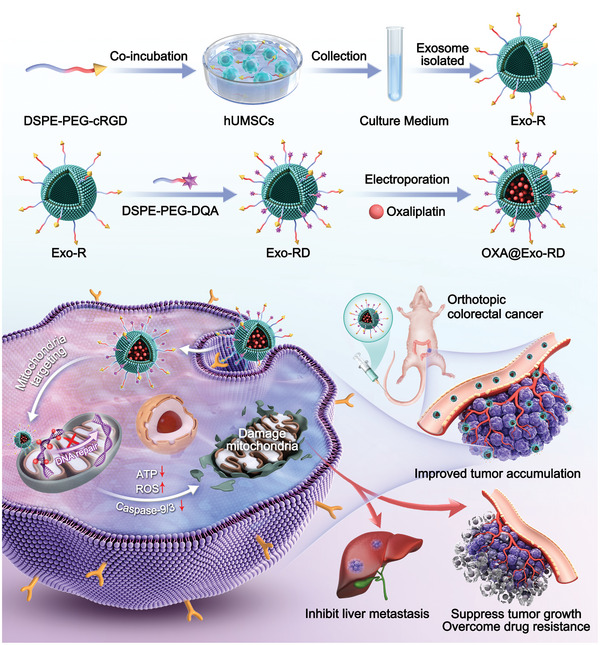
Schematic diagram for the construction of OXA@Exo‐RD nanocomposite and their applications in reversing chemoresistance and anti‐metastasis.

Herein, integrin ligands and mitochondrial affinity molecules modified exosomes were synthesized for encapsulating OXA to develop a sequentially targeted and mitochondrion‐dysfunctional nanodrug (OXA@Exo‐RD) (**Scheme** **1**). 1) The cRGD peptide on the surface of OXA@Exo‐RD conjugated with αvβ3 integrin receptor overexpressed in CRC cells to mediate cellular uptake and facilitate tumor accumulation. 2) Once OXA@Exo‐RD was internalized by tumor cells, the positive charge of DQA accumulated in mitochondria actuated by the high mitochondrial transmembrane potential, thereby increasing the local mitochondrial drug concentration. 3) Mitochondrial accumulation of DQA facilitated the overproduction of ROS, inhibited ATP production, and ultimately induced mitochondria‐mediated apoptosis and mitochondrial dysfunction. 4) The released OXA diffused into the mitochondrial matrix and interfered with mtDNA to exert anti‐tumor effects. 5) The mitochondrial damage resulted from OXA@Exo‐RD was hypothesized to not trigger the initiation of DNA repair, which can reduce the probability of chemoresistance. Given the critical role of mitochondria in regulating tumor growth and metastasis, OXA@Exo‐RD‐induced mitochondrial dysfunction could contribute to the enhanced anticancer effect against drug‐resistant CRC and inhibit CRC metastasis.

## 2 Results and Discussion

### 2.1 Synthesis and Characterization of OXA@Exo‐RD

To achieve mitochondrial‐targeted delivery of OXA to cancer cells, the mitochondriotropic molecule DSPE‐PEG_2000_‐DQA was first synthesized. The synthetic scheme was depicted in Figure S1 (Supporting Information). Subsequently, ^1^H‐NMR spectroscopy and MALDI‐TOF‐MS were employed to confirm the formation of DSPE‐PEG‐DQA. Figure S2 (Supporting Information) showed the ^1^H NMR spectra of DSPE‐PEG_2000_‐COOH, DQA, and DSPE‐PEG_2000_‐DQA. The characteristic peaks of DQA and DSPE‐PEG_2000_‐COOH were clearly present in the DSPE‐PEG_2000_‐DQA conjugate. After the conjugating reaction, the aromatic hydrogen signal that appeared at 6.8 ppm was attributed to DQA, which indicated the successful formation of DSPE‐PEG_2000_‐DQA.

Figures S3 and S4 (Supporting Information) showed the MALDI‐TOF‐MS spectra of DQA and DSPE‐PEG_2000_‐DQA. The MALDI‐TOF‐MS spectra of the product revealed the average mass of DSPE‐PEG‐DQA at m/z 3283.60, while that of DQA exhibited a sharp peak at m/z 455.20. The mass difference between DQA and DSPE‐PEG_2000_‐DQA was 2800 Da, which corresponded to the mass of DSPE‐PEG_2000_‐COOH. Therefore, the results of ^1^H‐NMR spectroscopy and MALDI‐TOF‐MS consistently confirmed the successful formation of DSPE‐PEG_2000_‐DQA.

**Figure 1:**
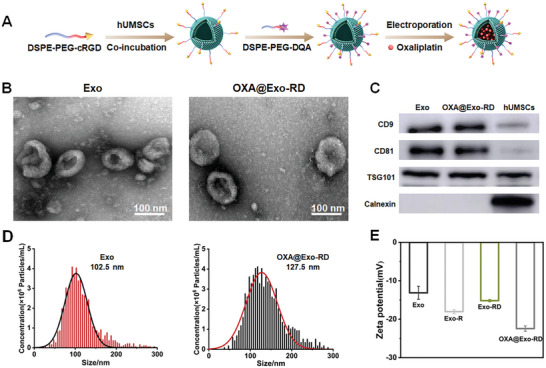
Characterization of OXA@Exo‐RD NPs. A) Schematic illustration of the preparation of OXA@Exo‐RD NPs. B) TEM images of Exo and OXA@Exo‐RD. C) The expression of exosomal biomarkers on Exo, OXA@Exo‐RD, and hUMSCs by western blotting analysis. D) Nanoparticle tracking analysis (NTA) of Exo and OXA@Exo‐RD. E) Zeta potentials of Exo, Exo‐R, Exo‐RD and OXA@Exo‐RD.

Exos were prepared using hUMSCs cells as donor cells. First of all, Exo‐R was purified from the culture supernatant, followed by conjugation with DQA through post‐insertion method to obtain Exo‐RD, which was finally encapsulated with OXA by electroporation to develop a sequentially targeted and mitochondrion‐dysfunctional drug delivery system OXA@Exo‐RD (**Figure** **1**A). The drug loading efficiency (DLE) of OXA in OXA@Exo‐RD was calculated to be 5.8%. As depicted in Figure 1B and Figure S5 (Supporting Information), the morphology of Exos and OXA@Exo‐RD by TEM measurements both exhibited a saucer shape, which was the typical morphology of Exos, indicating that Exos were successfully extracted and maintained their integrity after post‐modification and drug loading. The average diameter of OXA@Exo‐RD tested by NTA and DLS were 127.5 and 140.9 nm (Figure 1D; Figure S6, Supporting Information), which were slightly larger than those of unmodified Exos (102.5 and 126.0 nm). In addition, the surface potential of Exo‐R increased slightly from −18.0 ± 0.5 to −15.2 ± 0.3 mV after DQA surface modification, which may be attributed to the positive charge of the quaternary ammonium group of DQA. Meanwhile, the surface potential of Exo‐RD decreased to −22.4 ± 0.7 mV after OXA loading due to the inherent negative charge of OXA (Figure 1E). The changes in surface potential indirectly confirmed the successful surface modification and OXA loading. The fusion of exosomes and DSPE‐PEG‐DQA was observed by CLSM using DiI‐labeled Exo and FITC‐labeled‐DSPE‐PEG‐DQA. As shown in Figure S7 (Supporting Information), the red fluorescence overlapped well with the green fluorescence, and the colocalized yellow signal was obvious, further confirming the successful coating of the exosome. Western blotting results exhibited that both Exo and Exo‐RGD expressed Exo‐specific markers, including TSG101, CD9, and CD81, while negative expression of Calnexin, which was consistent with the biological characteristics of Exos (Figure 1C). The above results confirmed the successful construction of the sequential‐targeted drug delivery system. The drug release curve of OXA@Exo‐RD was studied under 4 °C (store condition) and 37 °C for 7 days. It was indicated that the leakage of OXA from OXA@Exo‐RD is less than 10% even over 7 days at 37 °C, confirming its excellent storage stability (Figure S8, Supporting Information). In addition, the particle size of OXA@Exo‐RD didn't exhibit obvious change over 7 days in DMEM containing 10% exosome‐depleted FBS and PBS, indicating its excellent physiological stability, which provides potential for subsequent in vitro and in vivo applications (Figure S9, Supporting Information).

### 2.2 In Vitro Dual‐Targeting Ability and Cytotoxicity Evaluation

**Figure 2:**
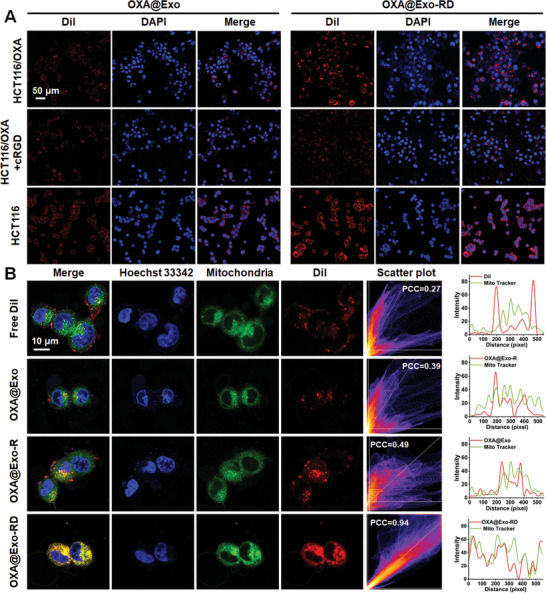
In vitro cellular uptake and mitochondrial targeting capability of OXA@Exo‐RD. A) Representative confocal images of HCT116/OXA or HCT116 cells incubated with DiI‐labeled OXA@Exo and OXA@Exo‐RD for 6 h. B) Representative confocal images demonstrating mitochondrial colocalization in HCT116/OXA cells after treatment with free DiI, DiI‐labeled OXA@Exo, DiI‐labeled OXA@Exo‐R, and DiI‐labeled OXA@Exo‐RD for 6 h. Hoechst 33342 (blue) and Mito–Tracker Green (green) were adopted to stain nuclei and mitochondria. The corresponding fluorescence intensity profiles of the merged images are illustrated in the right panel.

Given that the cRGD peptide is specifically conjugated with αvβ3 integrin receptor overexpressed in tumor cells. HCT116 cells and HCT116/OXA cells may have a higher uptake efficiency of OXA@Exo‐RD due to the overexpression of αvβ3 (Figures S11 and S12, Supporting Information). Therefore, the cellular uptake of DiI‐labeled OXA@Exo or OXA@Exo‐RD was first investigated by CLSM (**Figure** **2**A). As expected, HCT116/OXA cells incubated with OXA@Exo‐RD exhibited brighter red fluorescence than those incubated with OXA@Exo. Flow cytometry also demonstrated that OXA@Exo‐RD exhibited a higher cellular uptake compared with OXA@Exo (Figure S13, Supporting Information). After pretreatment with the receptor‐competitive molecule cRGD, the red fluorescence was significantly attenuated, indicating the excellent active targeting capacity and receptor‐dependent cellular uptake mechanism of OXA@Exo‐RD. In addition, the red fluorescence intensity of HCT116 cells upon OXA@Exo treatment was higher than that of HCT116/OXA cells, and the intensity difference was improved after OXA@Exo‐RD treatment. On one hand, the lipophilicity and positive charge of DQA were conducive to the adsorption and penetration of cell membrane. On the other hand, the surface modification of Exos evaded the recognition of P‐gp and thus reduced drug efflux.

We further examined the potential of these endocytosed nanoparticles to localize to the mitochondrial compartment through the colocalization of MitoTracker Green‐labeled mitochondria and DiI‐labeled nanoparticles. As illustrated in Figure 2B, the red fluorescence overlapped well with the mitochondrial green fluorescence after OXA@Exo‐RD treatment, and the colocalized yellow signal was evident. In sharp contrast, cells treated with DiI and DiI‐labeled OXA@Exo showed virtually negligible colocalization. In addition, the Pearson's colocalization coefficient (PCC) of the OXA@Exo‐RD group was 0.94, whereas the PCC of the free DiI and DiI‐labeled OXA@Exo groups were 0.28 and 0.39, respectively, further confirming the excellent mitochondrial targeting performance of OXA@Exo‐RD.

**Figure 3:**
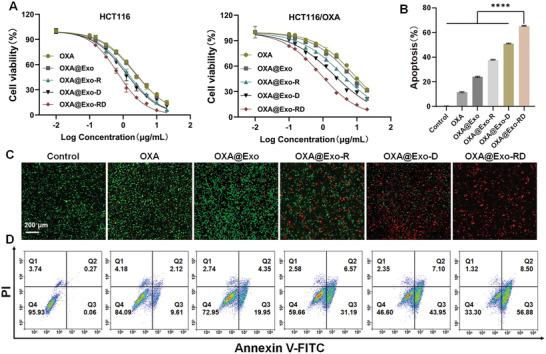
In vitro cytotoxicity evaluation. A) Anticancer activity analysis in HCT116 and HCT116/OXA cells after treatment with OXA, OXA@Exo, OXA@Exo‐R, OXA@Exo‐D, and OXA@Exo‐RD for 24 h. B) Apoptosis analysis of OXA, OXA@Exo, OXA@Exo‐R, OXA@Exo‐D, and OXA@Exo‐RD (4 µg mL^−1^ OXA) on HCT116/OXA cells. C) Representative confocal images of HCT116/OXA cells stained with calcein‐AM and PI following treatment with OXA, OXA@Exo, OXA@Exo‐R, OXA@Exo‐D, and OXA@Exo‐RD. D) Flow cytometry investigation of apoptosis following treatment with OXA, OXA@Exo, OXA@Exo‐R, OXA@Exo‐D, and OXA@Exo‐RD. The data are presented as the mean ± SD. ^****^ *p* < 0.0001 analyzed by one‐way ANOVA.

Increased cellular uptake and mitochondrial accumulation favored elevated mitochondrial DQA and OXA concentrations, ultimately accounting for enhanced therapeutic efficacy. The cytotoxicity of OXA@Exo‐RD was evaluated on OXA‐sensitive and OXA‐resistant HCT116 cells. As demonstrated in **Figure** **3**A, OXA@Exo‐RD showed stronger cytotoxicity to HCT116/OXA cells compared with OXA, as confirmed by the lowered IC_50_ index (0.87 vs 8.09 µg mL^−1^). In addition, the IC_50_ (resistance factor) ratio of HCT116/OXA to HCT116 decreased from 3.50 for OXA to 1.21 for OXA@Exo‐RD, indicating that OXA@Exo‐RD can alleviate OXA resistance. Later, the cell viability after different treatments was evaluated by Calcein‐AM/PI staining. As exhibited in Figure 3C, the red fluorescence signal representing dead cells in OXA@Exo‐RD‐treated group was the most prominent, indicating an excellent killing capability of OXA@Exo‐RD on HCT116/OXA cells. Annexin V‐FITC/PI staining was employed to further investigate the effect of OXA@Exo‐RD on cell apoptosis based on flow cytometry. Compared with cells treated with OXA (11.73%) and OXA@Exo‐D (51.05%), the apoptotic rate of cells treated with OXA@Exo‐RD was substantially increased (65.38%) (Figure 3B,D). In addition, CompuSyn analysis results showed that the combination index (CI) values for the combined treatment of OXA@Exo‐RD were all <1 in HCT116/OXA cells, indicating that OXA and DQA have synergistic antitumor effects in vitro (Figure S14, Supporting Information). Overall, the results of flow cytometry and live/dead staining consistently confirmed that OXA@Exo‐RD was an effective nanomedicine for inhibiting HCT116/OXA cells growth.

### 2.3 Mitochondrial Dysfunction and Mitochondrial‐Dependent Pathway

**Figure 4:**
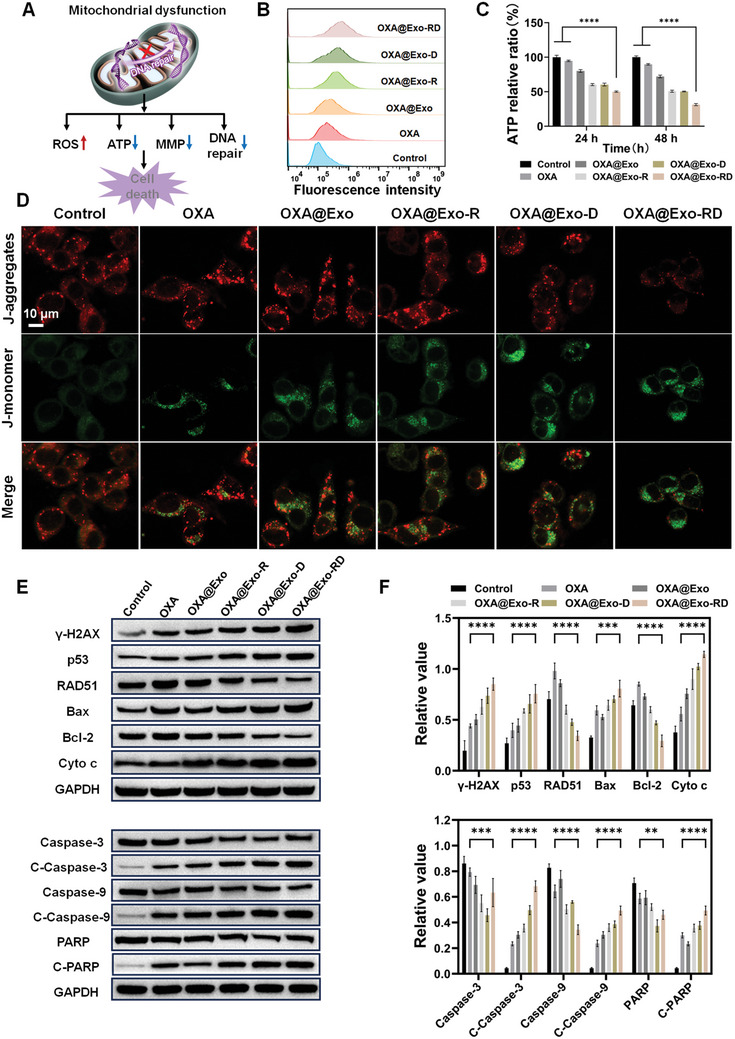
Study of mitochondrial dysfunction and mitochondrion‐dependent pathway. A) Schematic diagram of mitochondrial damage caused by OXA@Exo‐RD. ROS production B), and ATP depletion C) in HCT116/OXA cells treated with OXA, OXA@Exo, OXA@Exo‐R, OXA@Exo‐D, and OXA@Exo‐RD. D) Representative fluorescence images of MMP in HCT116/OXA cells after treatment with OXA, OXA@Exo, OXA@Exo‐R, OXA@Exo‐D, and OXA@Exo‐RD. E) Western blotting analysis of apoptosis‐related proteins expressions and F) protein‐to‐GADPH ratios in HCT116/OXA cells of different groups (*n* = 3). The data are presented as the mean ± SD. ^**^ *p* < 0.01, ^***^ *p* < 0.001, and ^****^ *p* < 0.0001 analyzed by two‐way ANOVA.

Given that mitochondria play a key role in modulating cellular function, increased accumulation of OXA and DQA in mitochondria may induce mitochondrial oxidative stress, eventually leading to mitochondrial dysfunction and cell death (**Figure** **4**A). Herein, flow cytometry was performed to assess the ROS level of HCT116/OXA cells using DCFH‐DA as a ROS indicator. As expected, abundant fluorescence was detected in HCT116/OXA cells following treatment with the sequentially targeted nanoparticle OXA@Exo‐RD compared with OXA@Exo, which was attributed to their higher cellular internalization (Figure 4B). In addition, MitoSOX Red was further adopted as an indicator to evaluate the mitochondrial ROS level using CLSM and a microplate reader. As shown in Figures S15 and S16 (Supporting Information), the fluorescence intensity of OXA@Exo‐RD significantly outweighed other treatment groups, which may be due to the increased accumulation of DQA and OXA mediating ROS generation and accumulation in mitochondria, while the enhanced ROS accumulation may further induce mitochondrial dysfunction.

Drug efflux occurs through energy‐dependent pathways. A decrease in intracellular ATP content can not only effectively reduce drug efflux but also enhance intracellular drug accumulation, thereby reducing drug resistance.^[^ ^42^ ^]^ Therefore, the ATP detection kit was employed to evaluate the effect of OXA@Exo‐RD on ATP content. As exhibited in Figure 4C, OXA@Exo‐RD treatment for 48 h resulted in a 68.7% reduction in ATP generation, which was higher compared to the OXA group (10.3%), confirming that OXA@Exo‐RD‐mediated ROS generation could further induce mitochondrial dysfunction, thereby effectively reducing the ATP content in HCT116/OXA cells.

Mitochondrial damage causes pro‐apoptotic factors release, which in turn initiates apoptosis. Since the change of MMP is related to the generation of ATP via oxidative phosphorylation and can be adopted to monitor mitochondrial biological activity. Herein, the change of MMP was monitored by JC‐1 staining to assess mitochondrial dysfunction. JC‐1 monomers exhibit green fluorescence when MMP is low, while JC‐1 aggregates demonstrate red fluorescence when MMP is high.^[^ ^43^ ^]^ In contrast to the control group, the red fluorescence in the OXA@Exo‐RD‐treated group was weakened while the green fluorescence was enhanced, indicating that OXA@Exo‐RD effectively decreased the MMP and impaired mitochondrial function (Figure 4D).

The above results confirmed its excellent capability of inducing cell apoptosis, and western blotting was subsequently adopted to further clarify the apoptotic pathway of OXA@Exo‐RD (Figure 4E,F). First of all, γ‐H2AX and p53 were adopted as DNA damage markers to assess the extent of DNA damage.^[^ ^44^, ^45^ ^]^ OXA@Exo‐RD treatment resulted in elevated expressions of γ‐H2AX and p53, indicating severe DNA damage in OXA‐resistant cells. Since RAD51 is a critical factor in the initiation of double‐strand DNA repair, the expression of RAD51 was also tested to determine whether OXA@Exo‐RD initiated DNA repair.^[^ ^46^, ^47^, ^48^ ^]^ The results revealed that OXA@Exo‐RD reduced the expression of RAD51 compared with the OXA‐treated group, indicating that OXA@Exo‐RD induced severe DNA damage and hindered the initiation of DNA repair. Mitochondrial outer membrane permeabilization (MOMP) is a signaling pathway that triggers cell death, which is regulated by the pro‐apoptotic protein Bax and the anti‐apoptotic protein Bcl‐2. p53 binds to Bcl‐2 and inhibits its expression, while the released Bax leads to increased MOMP.^[^ ^49^, ^50^ ^]^ We further detected the expression of associated proteins, and the results revealed that OXA@Exo‐RD boosted Bax expression while decreasing Bcl‐2 expression. As MOMP increases, intermembrane space (IMS) proteins (e.g., Cyto c) are released into the cytoplasm, triggering the caspase cascade reaction and causing Caspase‐9 and Caspase‐3 cleavage.^[^ ^51^, ^52^ ^]^ Upon OXA@Exo‐RD treatment, the expression of Cyto c enhanced, indicating that OXA@Exo‐RD activated subsequent cell apoptosis. Compared with other groups, OXA@Exo‐RD treatment resulted in the largest upregulation of C‐Caspase‐3, C‐Caspase‐9, and C‐PARP. The above results all indicated that the apoptotic mechanism triggered by OXA@Exo‐RD was strongly associated with the p53‐mediated mitochondrial pathway. Taken together, OXA and DQA released from mitochondria in situ amplified oxidative stress (mtROS), decreased MMP, resulting in mitochondrial damage and programmed cell death.

### 2.4 In Vitro Antimetastatic Effect

**Figure 5:**
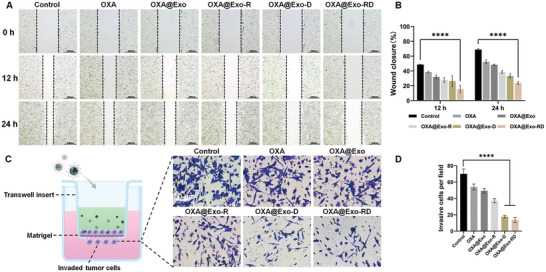
In vitro antimetastatic effect of OXA@Exo‐RD. A) Representative images illustrating in vitro wound healing assay and B) quantitative analysis of wound closure rate (*n* = 3). ^****^ *p* < 0.0001 analyzed by two‐way ANOVA. C) Schematic and photographs of transwell assay and D) corresponding invasive contents (*n* = 3). ^****^ *p* < 0.0001 analyzed by one‐way ANOVA. The data are presented as the mean ± SD.

Mitochondrial metabolism plays a key role in regulating tumor metastasis. As the cellular powerhouse, mitochondria generate ATP for cell activities such as cell migration and invasion.^[^ ^53^ ^]^ What is more, mitochondrial DNA mutations are involved in metastatic behavior change.^[^ ^54^ ^]^ The importance of mitochondria in tumor metastasis prompted us to examine the effects of constructs on cancer cell metastasis. First of all, the inhibitory effect of OXA@Exo‐RD on the migration of HCT116/OXA cells was assessed using the wound healing assay. After 24 h of incubation, significant wound healing occurred in both the control and OXA groups, while the wound closure of the OXA@Exo‐RD group was the lowest (76.3%), confirming their excellent suppression of HCT116/OXA cell migration (**Figure** **5**A,B).

The cell invasion was further verified by transwell assay using the Matrigel barrier. Different treatments decreased the motility of HCT116/OXA cells to some extent, while OXA@Exo‐RD minimized the invasion rate of HCT116/OXA cells across the barrier (Figure 5C,D), confirming their excellent inhibition on longitudinal movement and HCT116/OXA cells invasion. Overall, the above results highlighted that OXA@Exo‐RD increased tumor cell death and prevented tumor cell migration as well as reversed multidrug resistance, leading to an effective paradigm for reversing drug resistance and anti‐metastatic chemotherapy.

### 2.5 In Vivo Tumor Accumulation

**Figure 6:**
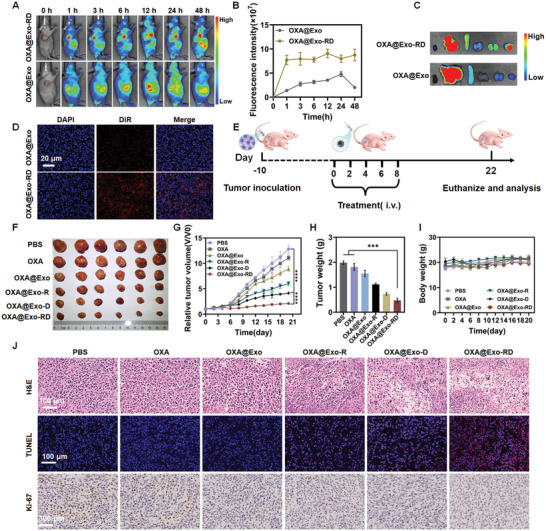
In vivo evaluation of tumor accumulation and anti‐tumor efficacy in resistant subcutaneous CRC. A) In vivo distribution of DiR‐labeled OXA@Exo, and OXA@Exo‐RD at predetermined times after intravenous administration via fluorescence imaging and B) the corresponding fluorescence intensity quantification of tumor sites (*n* = 3). C) Representative ex vivo fluorescence images of major organs and tumors acquired at 24 h post‐administration. D) DiR fluorescence distribution in tumor sections 24 h post‐administration. E) Schematic diagram of establishment of resistant subcutaneous CRC and the following treatment protocol. F) Photograph of the harvested tumors from each group. Change curves of G) tumor volume and I) body weight of mice after different treatments. H) Tumor weight of different groups at the endpoint of the study. J) Representative images of H&E, TUNEL, and Ki67 staining of tumor tissues from each group. The data are presented as the mean ± SD. ^***^ *p* < 0.001 and ^****^ *p* < 0.0001 analyzed by one‐way ANOVA.

Given the exceptional in vitro targeting capability of OXA@Exo‐RD, the tumor targeting behavior was further assessed through in vivo imaging. After intravenous injection of DiR‐labeled OXA@Exo or OXA@Exo‐RD, fluorescence images were obtained using the IVIS spectrum at specified time points. The fluorescence intensity of the tumor site in OXA@Exo‐RD‐treated mice was stronger than that in OXA@Exo‐treated mice during the monitored time range (**Figure** **6**A,B). Upon 24 h post administration, there was no appreciable fluorescence difference in the ex vivo liver, spleen, and lung between the different groups, while stronger fluorescence intensity at the tumor site was observed after coating dual‐targeting ligands (Figure 6C). Both in vivo and ex vivo imaging results confirmed its excellent tumor targeting effect. In addition, to further evaluate the tumor accumulation of different nanoparticles, confocal fluorescence imaging of tumor sections was performed. Compared with the OXA@Exo‐injected mice, stronger red fluorescence was observed upon OXA@Exo‐RD injection (Figure 6D), indicating increased tumor accumulation, which corresponds to the enhanced in vitro cellular uptake and in vivo fluorescence biodistribution. Taken together, OXA@Exo‐RD promoted tumor accumulation of drugs through dual targeting in vivo, which was expected to enhance antitumor activity and alleviate side effects on normal tissues.

### 2.6 In Vivo Anticancer Efficacy in Resistant Subcutaneous CRC

Encouraged by the excellent inhibitory efficacy of OXA@Exo‐RD against OXA‐resistant HCT116 cells, herein, we further assessed the in vivo anti‐tumor therapeutic efficacy in a subcutaneous tumor‐bearing model of OXA‐resistant CRC. The anti‐tumor efficacy was reflected by monitoring the changes in tumor volume in different treatment groups within 21 days (Figure 6E). Among all groups, OXA@Exo‐RD displayed the best growth inhibition effect on subcutaneous OXA‐resistant CRC with the smallest tumor volume (Figure 6F,G) and weight (Figure 6H). After treatment with OXA@Exo‐RD, no tumor‐bearing nude mice experienced significant weight loss, indicating negligible systemic toxicity (Figure 6I). To further assess the anti‐tumor efficacy from the perspective of pathology, tumor slices were stained with hematoxylin and eosin (H&E), terminal deoxynucleotidyl transferase‐mediated dUTP nick end labeling (TUNEL), and Ki‐67 for histological analysis. The largest apoptosis and necrosis areas, the least number of Ki‐67‐positive cells, and the highest number of TUNEL‐positive cells were observed in OXA‐resistant tumor slices upon OXA@Exo‐RD treatment (Figure 6J). The above histopathological results indicated that OXA@Exo‐RD significantly reduced tumor cell proliferation and enhanced tumor cell apoptosis, which was compatible with the efficacy study findings. Finally, the general toxicity test of the nanosystem showed that it did not affect the normal range of blood routines including white blood cell (WBC), red blood cell (RBC), hemoglobin (HGB), hematocrit (HCT), platelets (PLT), lymphocyte (Lymph), and blood biochemical indicators alanine transaminase (ALT), aspartate transaminase (AST), alkaline phosphatase (ALP), creatinine (CR), blood urea nitrogen (BUN), uric acid (UA) (Figures S18 and S19, Supporting Information). In addition, the results of blood biochemistry showed that different treatments had no obvious hepatic and kidney toxicity to mice. Furthermore, no appreciable histopathological changes were observed in the main organs of the heart, liver, spleen, lung and kidney (Figure S20, Supporting Information), demonstrating its biosafety and low toxicity for cancer therapy.

### 2.7 In Vivo Antitumor Efficacy in Resistant Orthotopic CRC

**Figure 7:**
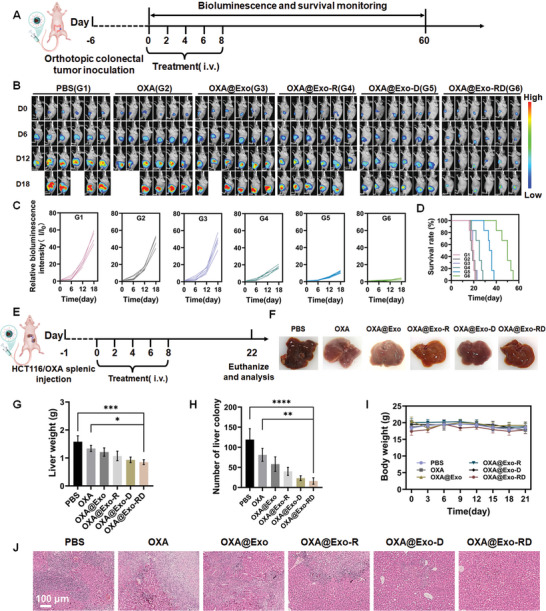
In vivo anti‐tumor and anti‐metastatic evaluation. A) Schematic diagram of establishment of resistant orthotopic CRC and the following treatment protocol. B) Bioluminescence images of mice bearing orthotopic CRC from each group and C) the corresponding bioluminescence intensity quantification of tumor sites (*n* = 6). D) Survival curves of the mice bearing orthotopic CRC in each group. E) Schematic diagram of establishment of liver metastasis model and the following treatment protocol. F) Representative images of liver after different treatments. G) Liver weight and H) number of liver colony of different groups at the endpoint of the study (*n* = 3). I) Changes of body weight after different treatments. J) Representative H&E‐stained images of liver tissues from each group. The data are presented as the mean ± SD. ^*^ *p* < 0.05, ^**^ *p* < 0.01, ^***^ *p* < 0.001, and ^****^ *p* < 0.0001 analyzed by one‐way ANOVA.

The antitumor efficacy of OXA@Exo‐RD was further evaluated using an orthotopic CRC model. Tumor burden was monitored employing the PerkinElmer IVIS spectrum according to the bioluminescent signals from luciferase‐expressing HCT116/OXA cells. The diagram of the treatment plan is illustrated in **Figure** **7**A. As depicted in Figure 7B,C, the bioluminescence signal of mice treated with OXA or OXA@Exo was moderately suppressed in comparison to the PBS group. Clearly, the mice treated with OXA@Exo‐RD exhibited the weakest bioluminescent signal, validating the preferable antitumor potency. At the same time, the survival of mice in different groups was monitored. Compared with the PBS group, the survival of mice in OXA and OXA@Exo groups remained nearly unchanged, while the survival was significantly prolonged after treatment with OXA@Exo‐D or OXA@Exo‐RD. Among them, 66% of the mice in the OXA@Exo‐RD group survived for at least 50 days (Figure 7D). Our experiments further demonstrated that OXA@Exo‐RD could effectively suppress the progression of orthotopic OXA‐resistant CRC and prolong the survival of mice.

### 2.8 In Vivo Anti‐Metastatic Evaluation

Liver metastasis is a primary cause of mortality in CRC. Given the critical role of mitochondria in cancer metastasis, we further investigated the anti‐metastatic potential of OXA@Exo‐RD in the HCT116/OXA metastatic model (Figure 7E). As illustrated in Figure 7F,G, the livers of mice treated with PBS exhibited diffuse metastatic lesions and the highest tumor burden based on liver weight. Unfortunately, OXA alone had a minimal impact on liver metastases. Among them, the number of liver metastases was the least after OXA@Exo‐RD treatment (Figure 7H). The inhibition rates of metastatic sites in the OXA, OXA@Exo, and OXA@Exo‐R groups were 31.9%, 51.2% and 66.3%, respectively, in comparison to the PBS group. In addition, the OXA@Exo‐D and OXA@Exo‐RD enhanced the inhibition rate to 80.7% and 86.6%, respectively, which were ≈1.5 and 1.7 times that of the OXA group, confirming excellent anti‐metastatic ability of OXA@Exo‐RD. Of particular note, no obvious weight loss of mice occurred during the whole experiment, indicating negligible systemic toxicity of OXA@Exo‐RD (Figure 7I). To further evaluate the metastasis inhibition efficacy from the perspective of pathology, tumor slices were stained with H&E (Figure 7J). In the livers of OXA@Exo‐RD‐treated mice, metastasized HCT116/OXA cells were reduced, whereas higher tumor burdens were observed in the OXA and OXA@Exo groups, implicating the excellent anti‐metastatic potential of OXA@Exo‐RD. Taken together, OXA‐encapsulated sequentially targeted nanoparticles may promote mitochondrial dysfunction, which could be a potential strategy for overcoming chemoresistance and reducing the occurrence of metastasis.

## 3 Conclusion

In summary, we developed a sequentially targeted and mitochondrion‐dysfunctional nanodrug (OXA@Exo‐RD) that simultaneously improves drug concentration and potency in the targeted organelle to overcome chemoresistance and reduce metastasis. By efficient targeting to mitochondria, OXA@Exo‐RD enhanced the spatiotemporal levels of DQA and OXA. Mitochondrial accumulation of DQA facilitated the overproduction of ROS, leading to mitochondria‐mediated apoptosis and mitochondrial dysfunction. Meanwhile, the released OXA not only damaged mtDNA to exert anti‐tumor effects but also avoided DNA repair initiation, thereby overcoming chemoresistance. Notably, OXA@Exo‐RD‐induced mitochondrial dysfunction exhibited excellent growth and metastasis inhibition against drug‐resistant CRC. Although further rigorous experiments are required to evaluate its long‐term safety and efficacy, we believe that this sequentially targeted exosome drug delivery system would provide new opportunities for the clinical treatment of CRC.

## 4 Experimental Section

### Materials

DSPE‐PEG_2000_‐cRGD and DSPE‐PEG_2000_‐COOH were acquired from Xi'an Ruixi Biological Technology Co., Ltd. Dequalinium (DQA) was bought from Sigma–Aldrich. Oxaliplatin, 1‐ethyl‐3‐(3‐(dimethylamino)propyl) carbodiimide hydrochloride (EDC) and N‐hydroxysuccinimide (NHS) were supplied from Macklin Biochemical Co., Ltd. ATP content detection kit was soured from Beyotime Biotechnology. Reactive oxygen species (ROS) assay kit and mitochondrial membrane potential assay kit were bought from Dojindo Molecular Technologies. TSG10, CD9, CD81, and Calnexin primary antibodies for western blotting were obtained from Abcam. γ‐H2AX, p53, RAD51, Bax, Bcl‐2, Cytochrome c, Caspase‐9, C‐Caspase‐9, Caspase‐3, C‐Caspase‐3, GAPDH, PARP, and C‐PARP primary antibodies were supplied from Cell Signaling Technology. DiI and DiR were purchased from ThermoFisher Scientific.

### Measurements

The chemical composition and molecular weight were confirmed by a proton nuclear magnetic resonance (^1^H NMR) spectrometer (AVANCE III 400 MHz, Bruker) and MALDI‐TOF‐MS analysis (AXIMA‐Assurance, Shimadzu). The morphology was investigated with transmission electron microscopy (TEM, Talos F200X G2, FEI). The concentration and size distribution of Exos were provided by nanoparticle tracking analysis (NTA, NanoSight NS300, Malvern). The fluorescence images were obtained by confocal laser scanning microscopy (CLSM, Stellaris 5, Leica).

### Cell Culture

Human CRC cell line HCT116 and human OXA‐resistant CRC cell line HCT116/OXA were purchased from Meisen Cell Technology Co., Ltd. Human umbilical cord mesenchymal stem cell line (hUMSCs) was supplied by National Stem Cell Translational Resource Center. HCT116 cells and HCT116/OXA cells were maintained in DMEM complete medium supplemented with 10% FBS and 1% penicillin‐streptomycin at 37 °C in 5% CO_2_. hUMSCs were cultured in α‐MEM complete medium supplemented with 10% FBS and 1% penicillin‐streptomycin at 37 °C in 5% CO_2_.

### Synthesis of DSPE‐PEG_2000_‐DQA

The synthetic route of DSPE‐PEG_2000_‐DQA was shown in Figure S1 (Supporting Information). Specifically, DSPE‐PEG_2000_‐COOH (50 mg, 0.0176 mmol), NHS (2.0 mg, 0.0174 mmol), and EDC‐HCl (6.8 mg, 0.036 mmol) were dissolved in 4 mL of chloroform, followed by three drops of triethylamine, and the reaction mixture was carried out in the dark at room temperature for 3 h. The course of the reaction was monitored using thin‐layer chromatography (TLC) on silica gel plates. DQA (9.2 mg, 0.0174 mmol) in DMSO (4 mL) was then added to DSPE‐PEG_2000_‐COO‐NHS and agitated for 1 h. After using rotary evaporation to remove the chloroform, 20 mL of DI water was added. The crude product was dialyzed against DI water for 72 h with 3000‐Da MWCO regenerated cellulose dialysis tubing to remove uncoupled reactants, and then the residue was freeze‐dried to yield dry powder. Following freeze drying, the lyophilized powder was dissolved in water and filtered via a 0.45 µm filter membrane. The liquid was subsequently freeze‐dried and the product was analyzed using ^1^H NMR spectrometer and MALDI‐TOF mass spectrometry.

### Preparation and Characterization of OXA@Exo‐RD

As before reported, RGD‐engineered exosomes (Exo‐R) were obtained from cell culture supernatants using gradient centrifugation.^[^ ^55^, ^56^ ^]^ Concisely, hUMSCs were maintained in DMEM supplemented with 10% Exo‐depleted FBS and 1% penicillin‐streptomycin for 24 h. To modify exosomes with cRGD peptide, the culture medium was replaced with DMEM containing DSPE‐PEG_2000_‐cRGD (100 µg mL^−1^) and further cultured for 24 h. When the cells reached 80–90% confluency, the culture medium was collected with a pipette. The obtained supernatant was centrifuged at 300 × g for 10 min, 2000 × g for 10 min, and 10 000 × g for 30 min, followed by sterile filtration using a 0.22 µm filter to remove cells and cell debris. Afterward, the resultant solution was ultracentrifuged at 110 000 × g for 90 min and then washed with PBS at 110 000 × g for another 90 min. Finally, the resulting Exo‐R was resuspended in PBS and stored at −80 °C for further analysis. All centrifugations were conducted at 4 °C.

According to previous work, DQA was incorporated into Exo‐R by the post‐insertion method.^[^ ^57^ ^]^ Briefly, DSPE‐PEG_2000_‐DQA was dissolved in HEPES buffer at 60 °C for 15 min to form micelles. After that, the Exo‐R suspension was mixed with the above solution at 40 °C for 3 h. After the crude products were cooled to room temperature, they were promptly purified using size exclusion chromatography to obtain cRGD and DQA co‐modified exosomes (Exo‐RD).

In order to encapsulate oxaliplatin (OXA) into Exo‐RD, electroporation was performed on an X‐Porator H1 electroporation system at 200 V and 100 µF. After that, the prepared OXA@Exo‐RD was collected by centrifugation and stored at 4 °C for further use.

To ascertain the drug loading efficiency (DLE) of OXA, the absorbance of different concentrations of OXA was first determined by a UV spectrophotometer to prepare a standard curve. After that, 1% Triton X‐100 was added to 10 µL of exosome solution to break the membrane, and the absorbance of OXA in OXA@Exo‐RD was measured at the same time. Finally, the DLE of OXA in OXA@Exo‐RD was calculated according to Equation (1).

(1) $$DLE\left(\%\right)=\frac{Weight\;of\;loadedOXA}{Weight\;of\;OXA@Exo-RD}\times 100\%$$

The presence of exosomal markers TSG101, CD9, CD81, and Calnexin was assessed using Western blot. The shape of exosomes was observed using TEM, while the concentration and particle size distribution were identified by NTA.

### Detection of Integrin αvβ3 Expression

FITC‐αvβ3 antibody LM609 was adopted to study the expression of integrin αvβ3 in HCT116 and HCT116/OXA cells. A portion of HCT116/OXA and HCT116 cells were seeded on confocal culture plates, and incubated with FITC‐αvβ3 antibody for 30 min. After washing three times with PBS, the cells were then stained with TRITC phalloidin for the cytoskeleton and DAPI for nuclei, and finally, the stained cells were observed with CLSM. The other portion of HCT116 and HCT116/OXA cells in the logarithmic growth phase were collected, followed by incubation with FITC‐αvβ3 antibody LM609 and negative control antibody FITC‐IgG1 for 30 min. Finally, the content of integrin αvβ3 was analyzed by flow cytometry.

### Cellular Uptake Assay In Vitro

To study the influence of targeted group modification on cell uptake behavior, OXA@Exo‐RD was labeled with 1,1′‐dioctadecyl‐3,3,3′,3′‐tetramethylindocyanine perchlorate (DiI) per the manufacturer's protocol. HCT116 and HCT116/OXA cells were cultured in confocal culture dishes and treated with DiI‐labeled exosomes for 6 h. Afterward, the nuclei were stained using Hoechst 33342. Finally, CLSM was used to evaluate the internalization of DiI‐labeled OXA@Exo‐RD. Flow cytometry was further used to evaluate cellular uptake. Specifically, HCT116/OXA cells were first treated with DiI‐labeled OXA@Exo and OXA@Exo‐RD for 6 h. Afterward, the HCT116/OXA cells were washed, digested, and collected for flow cytometry analysis to quantitatively detect the cellular uptake.

### IC_50_ Assay

The CCK‐8 assay was carried out to determine the half inhibitory concentration (IC_50_) of OXA. First of all, HCT116 and HCT116/OXA cells were seeded into 96‐well plates (1 × 10^4^ per well) and cultured overnight. Then, OXA, OXA@Exo, OXA@Exo‐R, OXA@Exo‐D, and OXA@Exo‐RD containing 0.01–20 µg mL^−1^ of Pt were added to the plate and incubated for 24 h. After adding 10 µL of CCK‐8 solution to each well for 2 h incubation, and the absorbance at 450 nm was recorded with a microplate reader. The IC_50_ was calculated by the dose‐response curves after consecutive dilution of different agents.

### Intracellular ROS Detection

ROS production in HCT116/OXA cells was detected using 2',7'‐dichlorofluorescein diacetate (DCFH‐DA). HCT116/OXA cells were seeded in 6‐well plates (5 × 10^5^ per well) and incubated for 24 h and then cultured in a medium containing OXA, OXA@Exo, OXA@Exo‐R, OXA@Exo‐D, and OXA@Exo‐RD (equivalent OXA dose of 4 µg mL^−1^) for 4 h. Next, DCFH‐DA (20 µm) was introduced and incubated for 10 min after being washed three times with PBS. Finally, the cells were harvested to analyze the generation of ROS using flow cytometry.

### MitoROS Generation Detection

MitoSOX Red mitochondrial superoxide indicator was adopted to evaluate the intracellular MitoROS level. First of all, HCT116/OXA cells were treated with OXA, OXA@Exo, OXA@Exo‐R, OXA@Exo‐D, and OXA@Exo‐RD (equivalent OXA dose of 4 µg mL^−1^) for 4 h. Next, MitoSOX Red (5 µm) was introduced and incubated for 10 min after being washed three times with PBS. Afterward, a portion of cells were collected and the fluorescence intensity was measured using a microplate reader. The other portion of cells were stained with DAPI for nuclei and observed under a CLSM.

### ATP Content Analysis

HCT116/OXA cells were seeded in six‐well plates (5 × 10^5^ per well) and incubated for 24 h. Different wells were treated with fresh culture medium containing OXA, OXA@Exo, OXA@Exo‐R, OXA@Exo‐D, and OXA@Exo‐RD (equivalent OXA dose of 4 µg mL^−1^) for 24 and 48 h, respectively. Subsequently, the HCT116/OXA cells were harvested to detect the ATP levels in each group using an ATP content detection kit.

### Apoptosis Pathway Analysis

Western blotting was adopted to explore the mechanism of apoptosis produced by OXA@Exo‐RD in HCT116/OXA cells. HCT116/OXA cells were seeded in six‐well plates (5 × 10^5^ per well) and incubated for 24 h. The cells were then treated with OXA, OXA@Exo, OXA@Exo‐R, OXA@Exo‐D, and OXA@Exo‐RD (equivalent OXA dose of 4 µg mL^−1^) for another 24 h. Each group of equivalent proteins was separated by SDS‐PAGE gel electrophoresis and transferred to a PVDF membrane under standard procedures. After being blocked with 5% BSA solution for 2 h, the membranes were then subjected to overnight incubation at 4 °C with different primary antibodies, followed by incubation with secondary antibodies at room temperature for 2 h to label the target protein.

### Mitochondria‐Targeting Localization

CLSM was used to evaluate the mitochondrial colocalization of different DiI‐labeled nanoparticles in HCT116/OXA cells. HCT116/OXA cells were seeded in confocal glass‐bottomed dishes and treated with free DiI, DiI‐labeled OXA@Exo, DiI‐labeled OXA@Exo‐R, and DiI‐labeled OXA@Exo‐RD at a final DiI concentration of 1 µm for 6 h. After three washes with PBS, the cells were then stained with Mito‐Tracker Green and Hoechst 33343 dye for 30 min at 37 °C. Finally, the stained cells were viewed with a CLSM.

### Changes in Mitochondrial Membrane Potential (MMP)

The cationic dye JC‐1 was adopted to evaluate the loss of the MMP. First of all, HCT116/OXA cells were seeded in confocal culture dishes and incubated overnight. Following treatment with OXA, OXA@Exo, OXA@Exo‐R, OXA@Exo‐D, and OXA@Exo‐RD (equivalent OXA dose of 4 µg mL^−1^), cells were then treated with JC‐1 dissolved in DMEM for 20 min. After being washed with PBS, the fluorescence images were finally taken with CLSM.

### Live/Dead Cell Staining

The distribution of live and dead cells after different treatments was evaluated using calcein‐AM and PI dual staining. HCT116/OXA cells seeded in confocal glass‐bottomed dishes were cultured with OXA, OXA@Exo, OXA@Exo‐R, OXA@Exo‐D, and OXA@Exo‐RD (equivalent OXA dose of 4 µg mL^−1^) for 24 h. Live and dead cells were then stained with calcein‐AM and PI per the manufacturer's protocol. Finally, the fluorescence images of the stained cells were taken with CLSM.

### Apoptosis Assay

Flow cytometry was employed to detect cell apoptosis. HCT116/OXA cells seeded in six‐well plates were cultured with OXA, OXA@Exo, OXA@Exo‐R, OXA@Exo‐D, and OXA@Exo‐RD (equivalent OXA dose of 4 µg mL^−1^) for 24 h. The cells were then harvested and re‐suspended in PBS, followed by staining with Annexin V‐FITC/PI for 15 min. Untreated and single‐stained with Annexin V‐FITC or PI cells were used to adjust fluorescence compensation.

### Combination Index (CI) Analysis

The CompuSyn method was adopted to determine whether OXA and DQA in OXA@Exo‐RD have synergistic antitumor effects in vitro. First, the CCK‐8 kit was used to evaluate the cytotoxicity of Exo‐RD, OXA@Exo‐R, and OXA@Exo‐RD on HCT116/OXA cells, and the dose‐effect curve was drawn. Subsequently, the obtained parameters were input into CompuSyn software to calculate the CI value. The antitumor synergism was determined by the CI value, with CI values <1, = 1, and >1 indicate synergism, additive effect, and antagonism, respectively.

### Migration and Invasion Assays

The migration of HCT116/OXA cells was assessed using wound‐healing assay. Briefly, the monolayer cells seeded in 6‐well plates were carefully wounded using a 200 µL pipette tip and treated with different samples (OXA, OXA@Exo, OXA@Exo‐R, OXA@Exo‐D, and OXA@Exo‐RD). After incubation for 24 h, the cell migration of wound area was photographed on an inverted fluorescence microscope at 0, 12, and 24 h and the wound closure was calculated by Image J software.

Cell invasion was evaluated using a transwell system. Specifically, the transwell upper chamber was pre‐coated with 80 µL of diluted Matrigel (1:8 dilution, Corning) for 3 h. Then, 100 µL of serum‐free medium containing HCT116/OXA cells (4 × 10^4^ cells) was inoculated in the upper chamber, followed by 500 µL of complete medium adding to the lower chamber. After that, several solutions including OXA, OXA@Exo, OXA@Exo‐R, OXA@Exo‐D, and OXA@Exo‐RD (equivalent OXA dose of 4 µg mL^−1^) were added to the upper chamber and incubated for 24 h. After being fixed with methanol for 20 min, the invaded cells were further stained with 0.1% crystal violet for 10 min. Finally, the cells were counted and images were captured under an inverted fluorescence microscope.

### Establishment of Luciferase‐Expressing HCT116/OXA Cells

In 10 cm dishes, 293T cells were infected with lentiviral vectors containing the firefly luciferase gene as well as packaging vectors psPAX2 and pMD2.G with the aid of Lipofectamine 2000 per the manufacturer's instructions. The viral particle‐containing supernatant was collected after 48 h, followed by filtration with a 0.45 µm filter and added to HCT116/OXA cells. Uninfected cells were eliminated using 2 µg mL^−1^ puromycin.

### Animal Care and Subcutaneous Model Construction

Four to six weeks old female BALB/c nude mice used in animal experiments were acquired from Vital River (Beijing, China). Mice were raised in an animal facility with constant environmental conditions, including room temperature of 22 ± 2 °C, relative humidity of 50–70%, and a 12 h light‐dark cycle. Water and food for animals were free access. All animal procedures were carried out following the protocol approved by the Institutional Animal Care and Use Committee of Jinke Bona Biotech Co. (GENINK‐20240008, China).

To obtain subcutaneous tumor‐bearing mice, HCT116/OXA cells (5 × 10^6^ cells) were subcutaneously injected into the right hind limb of the BALB/c nude mice. The mice weight and tumor volume were recorded every two days and calculated using the formula: V = (length × width^2^)/2, where width and length represent the minimum and maximum diameters of the tumor. Once the tumor volume reached either 100 or 500 mm^3^, the mice were employed for tumor treatment assays and in vivo targeting assays, respectively.

### In Vivo Targeting Validation

To evaluate the in vivo targeting ability, the lipophilic fluorescent dye DiR was adopted to designate OXA@Exo‐RD and OXA@Exo. BALB/c nude mice were randomized into two groups and injected intravenously with DiR‐labeled OXA@Exo‐RD or OXA@Exo (DiR dose with 20 µg per mouse), respectively. Then, fluorescence images were obtained via the PerkinElmer IVIS spectrum at various time points (0, 1, 3, 6, 12, 24, and 48 h). The main organs and tumors were collected for ex vivo imaging at 24 h. Following imaging, the tumors were harvested, sliced and stained for cell nuclei.

### In Vivo Treatment of Subcutaneous CRC

BALB/c nude mice bearing HCT116/OXA tumors were randomly assigned to six groups (*n* = 6) and intravenously injected with PBS, OXA, OXA@Exo, OXA@Exo‐R, OXA@Exo‐D, and OXA@Exo‐RD at an OXA‐equivalent dose of 2 mg kg^−1^ every other day for five times. After 21 days, the tumors were harvested, fixed with 4% paraformaldehyde and embedded in paraffin. Later, paraffin‐embedded tumors were cut into 4 µm slices and then stained with hematoxylin and eosin (H&E) for histopathological analysis. For the therapeutic efficacy of tumor tissues, the tumor slices were stained with the TUNEL kit. In addition, Ki‐67 staining was adopted to verify tumor proliferation. Specifically, the tumor sections were deparaffinized, hydrated, immersed in antigen retrieval solution and boiled for antigen repair, followed by blocking endogenous peroxidase with 3% H_2_O_2_. The tumor sections were then incubated with anti‐Ki‐67 antibody at 4 °C overnight. Subsequently, the tumor sections were washed in PBS and incubated with HRP‐labeled secondary antibody at 37 °C for 30 min. After washing with PBS, the sections were developed with 3,3'‐diaminobenzidine (DAB) solution and counterstained with hematoxylin. Finally, the images were captured under an inverted fluorescence microscope.

### In Vivo Biosafety Assessment

The histological, hematological, and biochemical studies were employed the in vivo biosafety. Specifically, HCT116/OXA tumor‐bearing BALB/c nude mice were euthanized at the end of the treatment. Main organs including heart, liver, spleen, lung, and kidney were harvested, fixed with 4% paraformaldehyde and embedded in paraffin. Later, paraffin‐embedded tissues were cut into 4 µm sections and then stained with H&E. Whole blood samples from different groups were collected after 21 days of treatment, and an automatic hematology analyzer was used to evaluate hematological parameters. The whole blood samples were centrifuged to obtain serum, and the blood biochemical indicators were detected by an automatic biochemistry analyzer. The blood biochemical indicators included liver function indicators aspartate transaminase (AST), alanine transaminase (ALT), and alkaline phosphatase (ALP) as well as renal function indicators such as blood urea nitrogen (BUN), creatinine (CR), and uric acid (UA).

### In Vivo Treatment of Orthotopic CRC

In addition to the subcutaneous CRC model, an orthotopic CRC model was also established to further evaluate the therapeutic effect. First, 5 × 10^6^ luciferase‐expressing HCT116/OXA (HCT116/OXA‐Luc) cells were injected subcutaneously into the right hind limb of the mice to construct the subcutaneous tumor model. When the tumor volume reached 500 mm^3^, the tumor was cut into 2 mm^3^ fragments and sutured onto the intestine of BABL/C nude mice to construct the orthotopic CRC model.^[^ ^58^ ^]^ Six days later, the mice were randomly assigned to six groups (*n* = 6) and intravenously injected with PBS, OXA, OXA@Exo, OXA@Exo‐R, OXA@Exo‐D, and OXA@Exo‐RD at an OXA‐equivalent dose of 2 mg kg^−1^ every other day five times. To evaluate the therapeutic effect, mice were intraperitoneally injected with 100 µL of 15 mm AkaLumine‐HCl substrate on days 0, 6, 12, and 18, respectively, with an exposure time of 60 s. Finally, bioluminescence imaging of treated mice was performed using a PerkinElmer in vivo imaging system.

### In Vivo Anti‐Liver Metastasis Effect Evaluation

For the liver metastasis model, the spleen was tied and divided into two sections, each with an intact vascular pedicle. After that, 2 × 10^6^ HCT116/OXA cells in 50 µL DMEM were injected into the distal section of the spleen. The spleen was gently massaged with a cotton swab at the injection site for 5 min to avoid tumor cell extravasation and promote tumor cell migration into the liver. Five min after inoculation, when tumor cells had entered the portal vein, the hemi‐spleen containing the injected cells was excised to simulate primary tumor excision. On the day following splenic injection, the mice were randomly assigned to 6 groups (*n* = 3) and intravenously injected with PBS, OXA, OXA@Exo, OXA@Exo‐R, OXA@Exo‐D, and OXA@Exo‐RD at an OXA‐equivalent dose of 2 mg kg^−1^ every other day five times. The weight and activity of mice were recorded during the administration. At last, the mice were euthanized on day 21 and the liver metastatic sites were counted to assess the CRC liver metastatic levels.

### Statistical Analysis

Statistical data were analyzed using GraphPad Prism 10 software. All results were present as means ± standard deviation. The data's statistical significance was determined using one/two‐way analysis of variance (ANOVA). ^*^ *p* < 0.05, ^**^ *p* < 0.01, ^***^ *p* < 0.001, ^****^ *p* < 0.0001.

## Conflict of Interest

The authors declare no conflict of interest.

## Supporting information



Supporting Information

## Data Availability

The data that support the findings of this study are available from the corresponding author upon reasonable request.
