# Supplementary material for: A Mitochondria‐Targeted Biomimetic Nanomedicine Capable of Reversing Drug Resistance in Colorectal Cancer Through Mitochondrial Dysfunction
Source: Adv Sci (Weinh). 2025 Feb 12;12(13):2410630. doi: 10.1002/advs.202410630 (PMC11967798; doi:10.1002/advs.202410630)
Supplement: Supplementary file 1 — Supporting Information [file ADVS-12-2410630-s001.docx]

Supporting information

**A mitochondria-targeted biomimetic nanomedicine capable of** **reversing drug resistance in colorectal cancer through mitochondrial dysfunction**

Xiaohui Wang^1,2^, Zhiyuan Xu^4^, Jian Wang^1^, Chunrong Wu^1^, Lin Zhang^5^, Chengyuan Qian^1^, Yang Luo^3^*, Yanjuan Gu^2^*, Wing-Tak Wong^2^*, and Debing Xiang^1^*

**Supplementary figures**


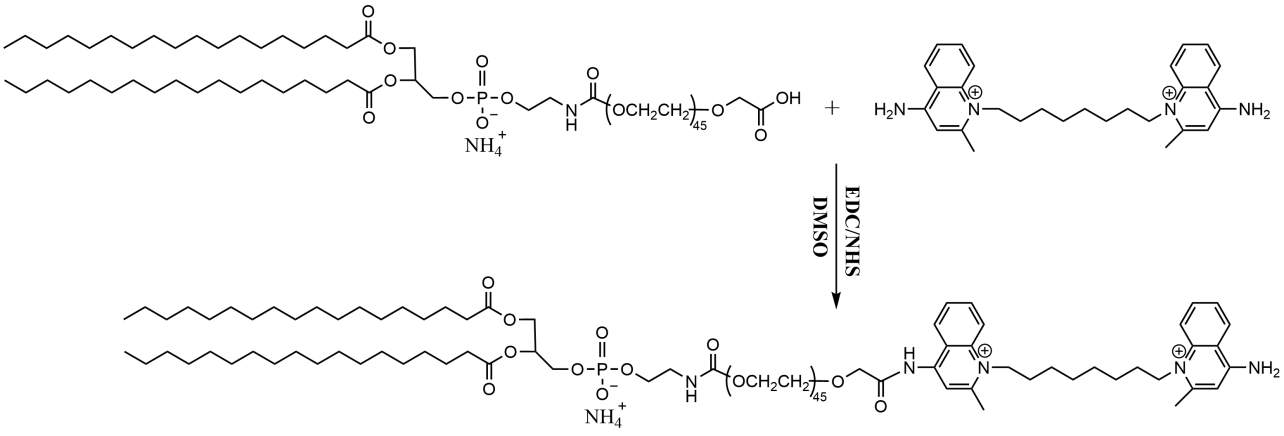


Figure S1 Synthetic scheme for DSPE-PEG_2000_-DQA conjugate.


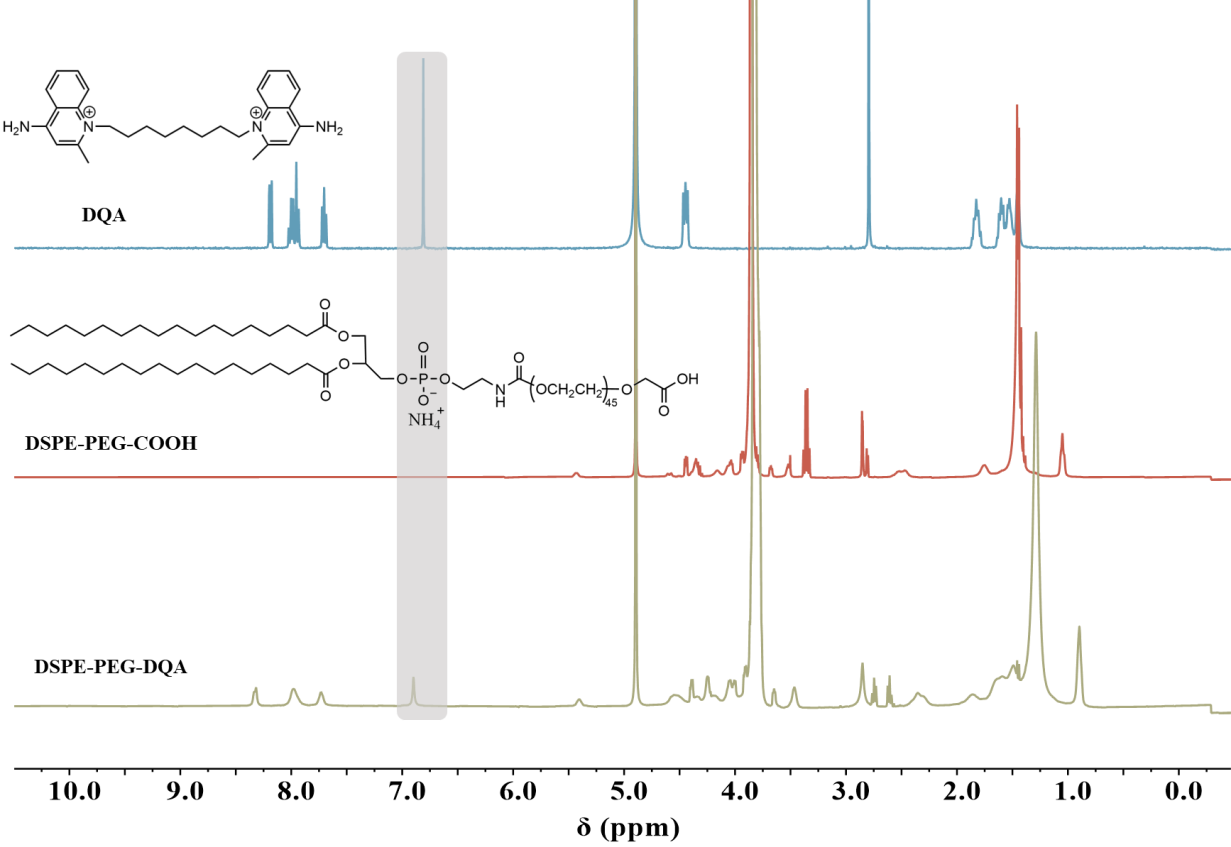


Figure S2 ^1^H NMR spectra of DQA, DSPE-PEG_2000_-COOH, and DSPE-PEG_2000_-DQA conjugate (D_2_O, 400 MHz).


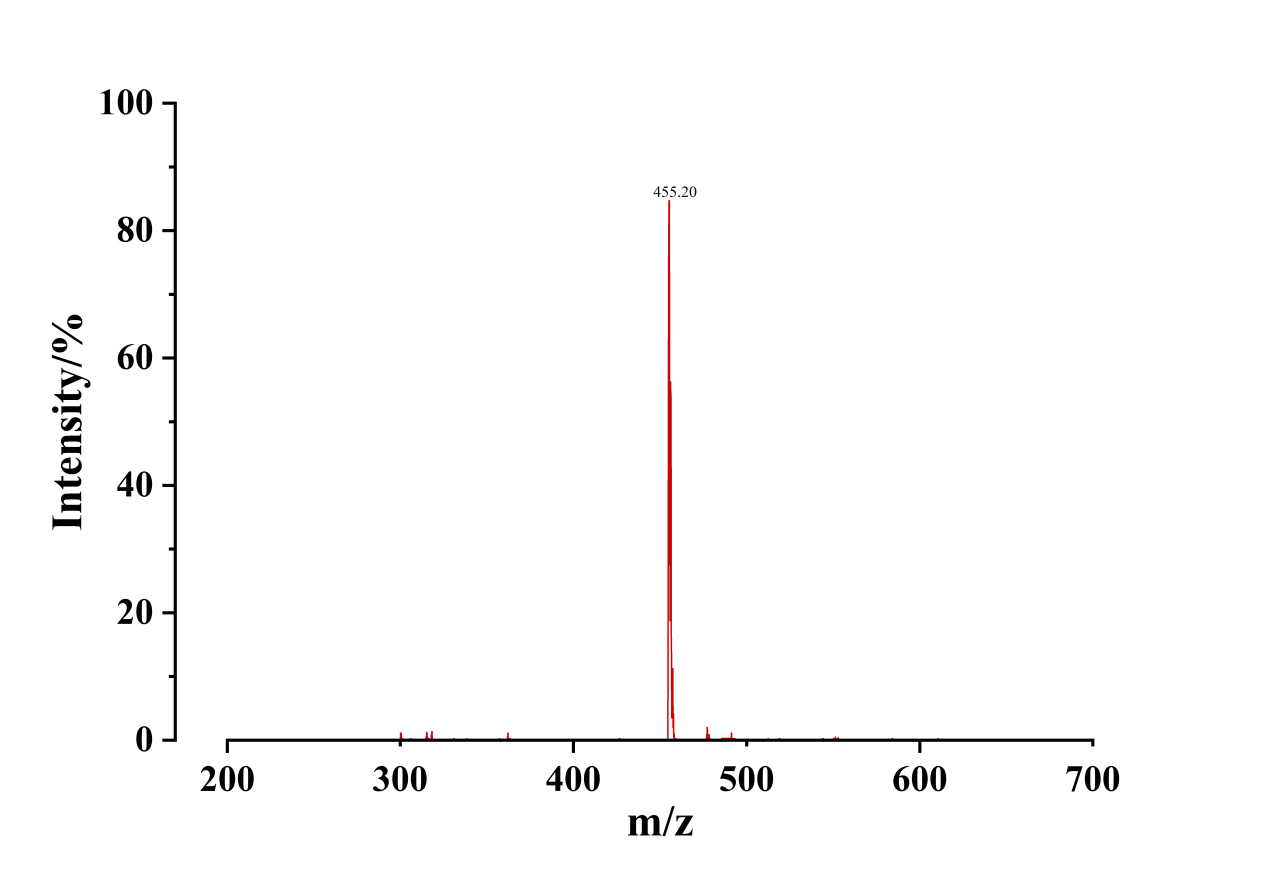
 Figure S3 MALDI-TOF-MS spectra of DQA.


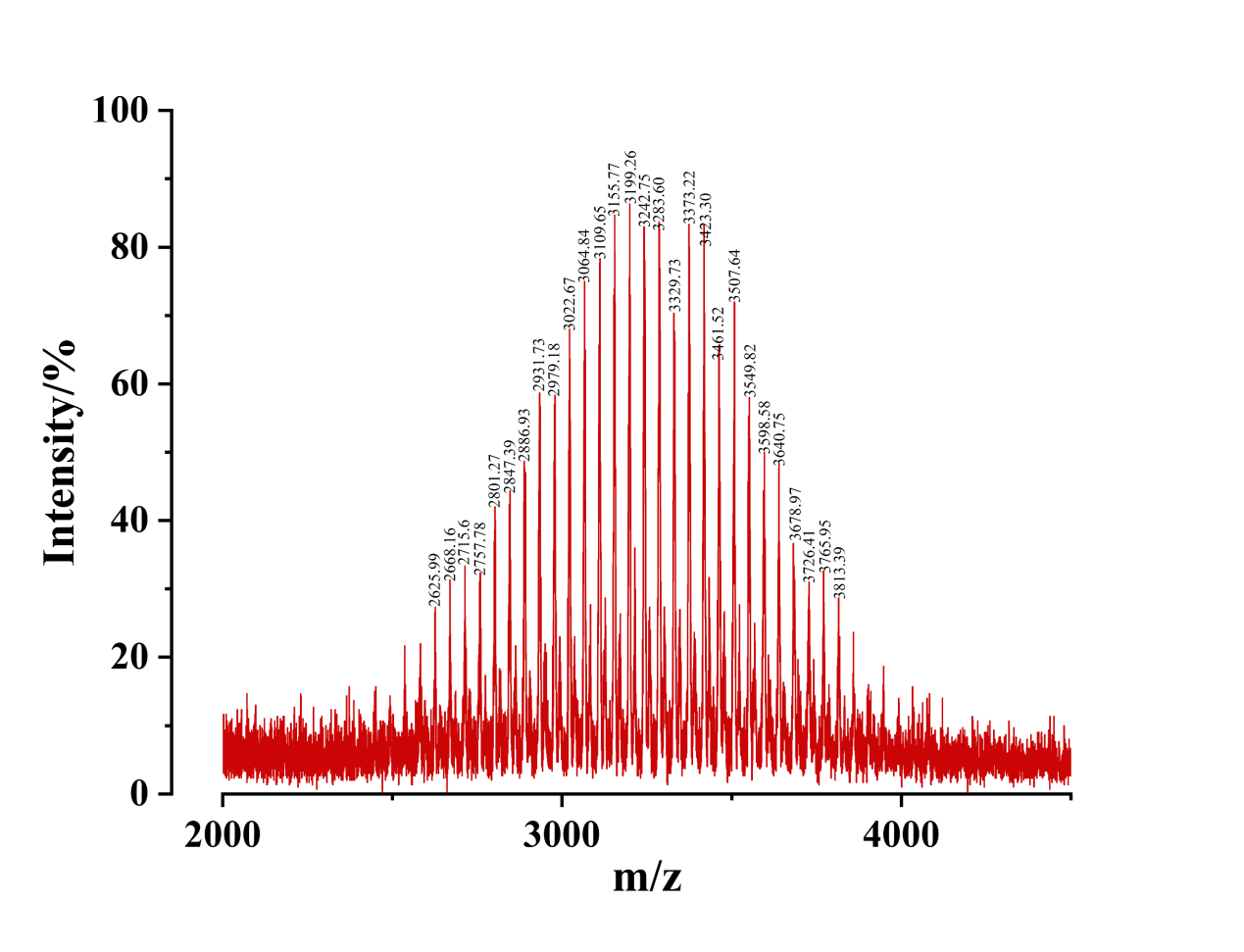
 Figure S4 MALDI-TOF-MS spectra of DSPE-PEG_2000_-DQA.


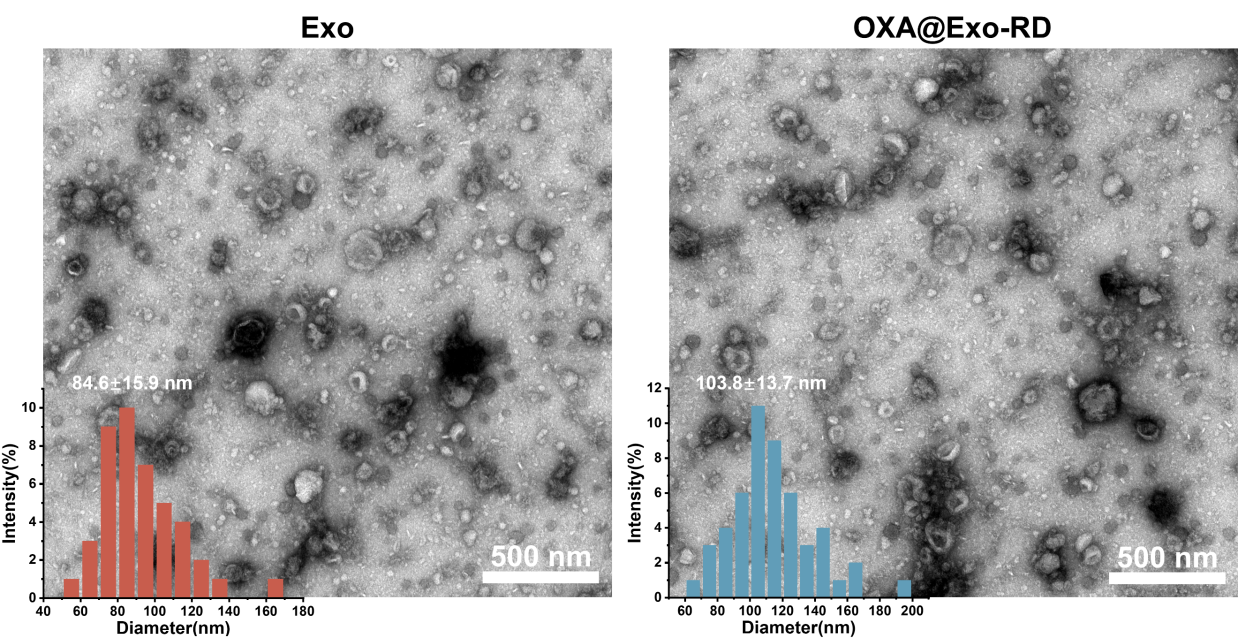


Figure S5 TEM image with size distribution of Exo and OXA@Exo-RD.


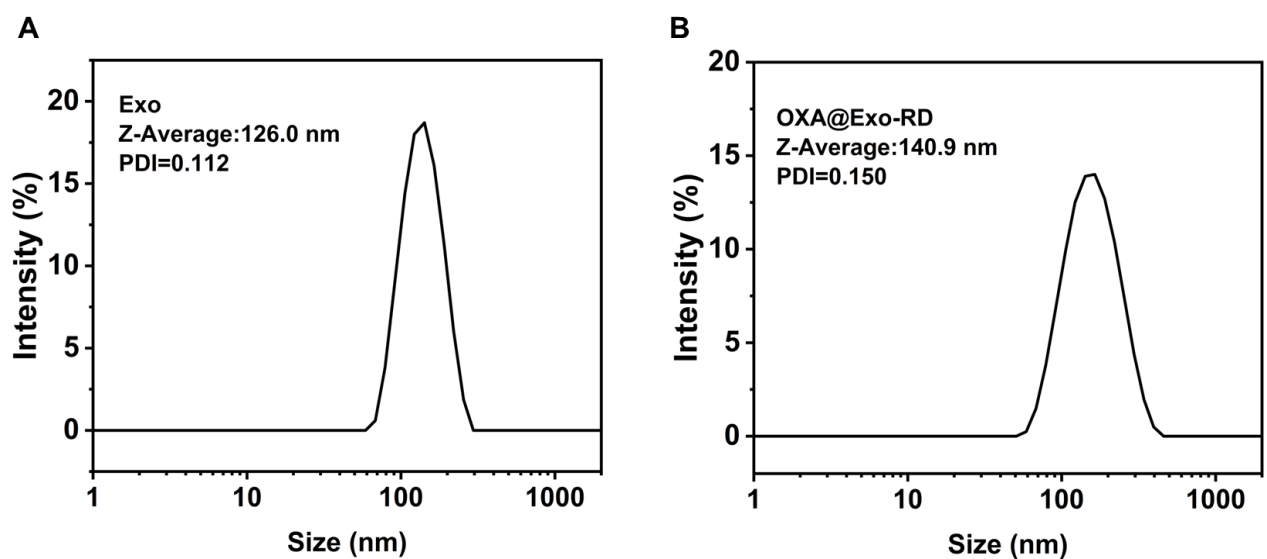


Figure S6 DLS size distribution and the polydispersity index of Exo (A) and OXA@Exo-RD (B).

**
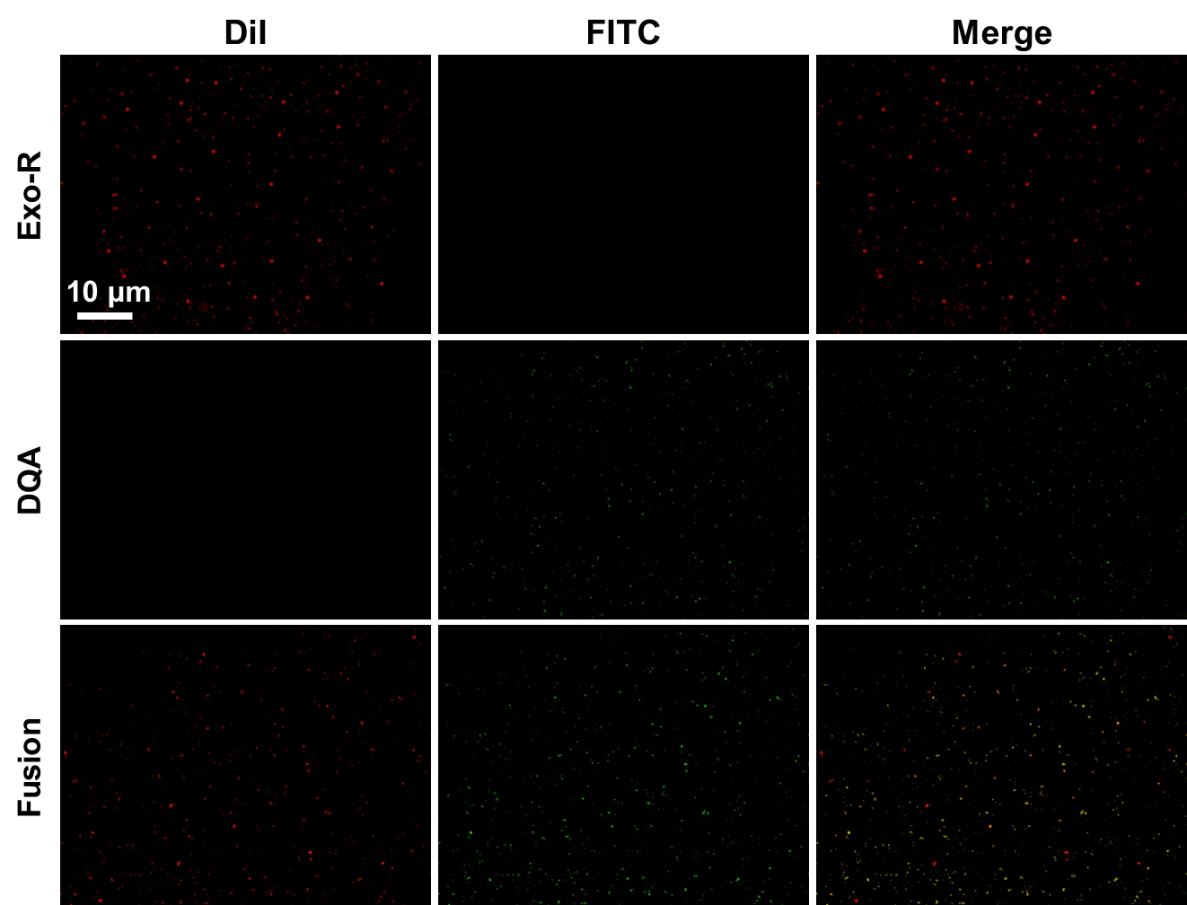
**

Figure S7 Confocal images showed the fusion of DiI-Exo-R and DSPE-FITC-PEG-DQA after 3 h incubation.


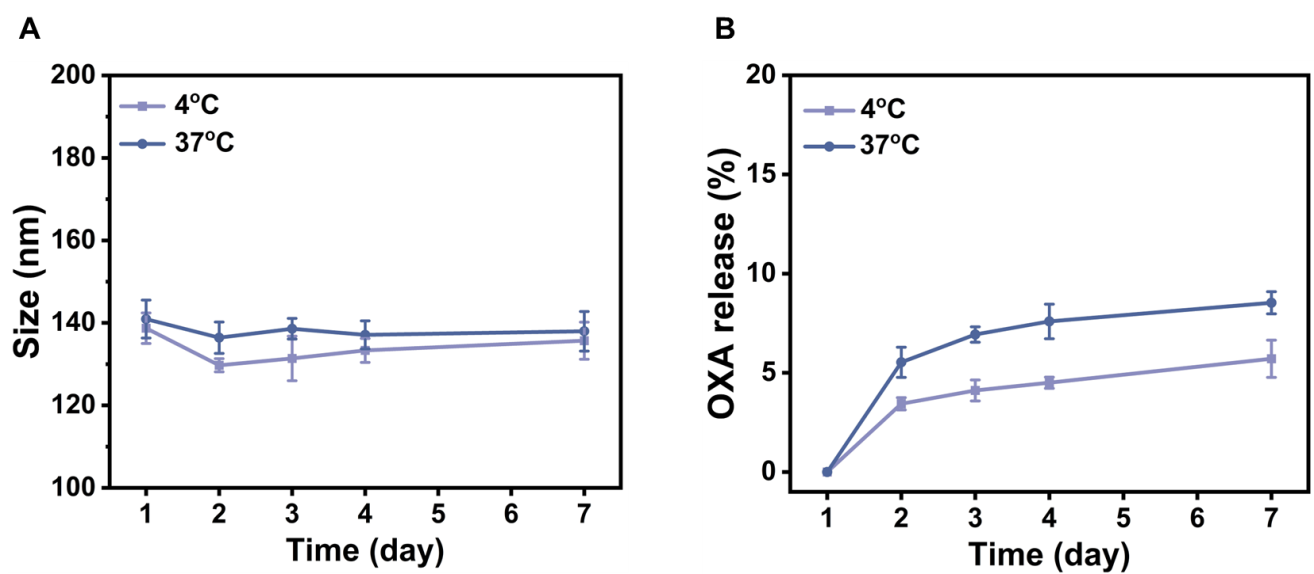


Figure S8 DLS measurement of size change of OXA@Exo-RD (A) and release profile of OXA from OXA@Exo-RD (B) at different temperature over a period of 7 days.


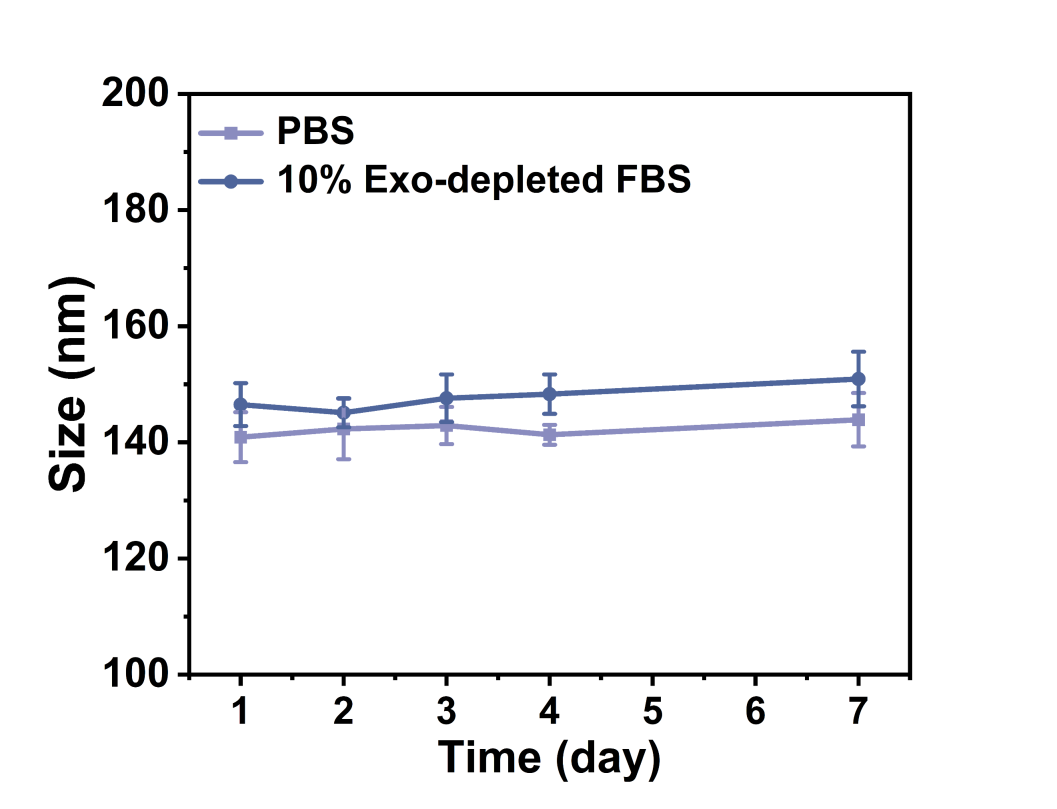


Figure S9 The change of particles size of OXA@Exo-RD meaured by DLS after incubated in DMEM medium with 10% exosome-depleted FBS and PBS for 7 days.


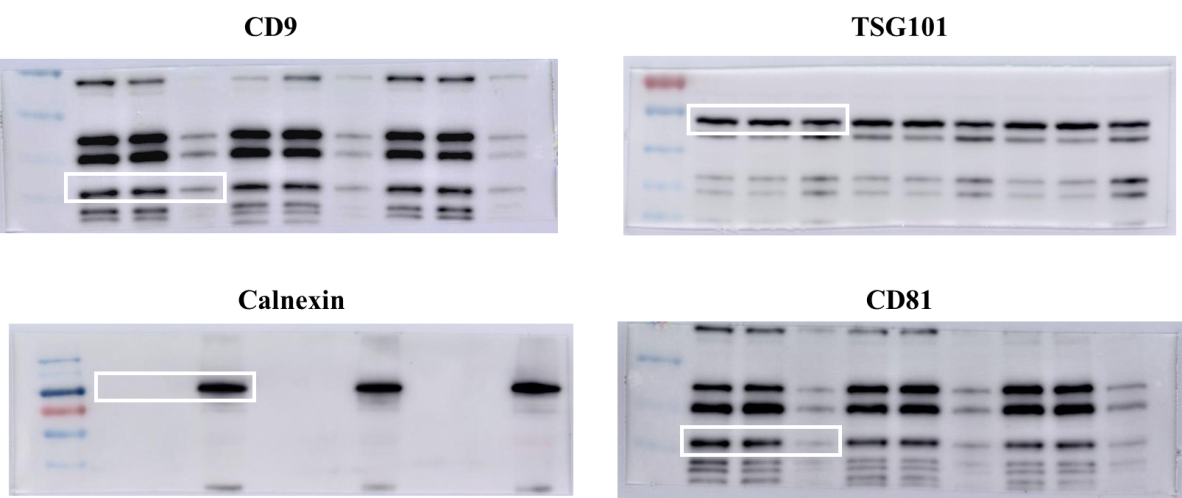


Figure S10 Raw data of exosomal biomarkers using western blotting.


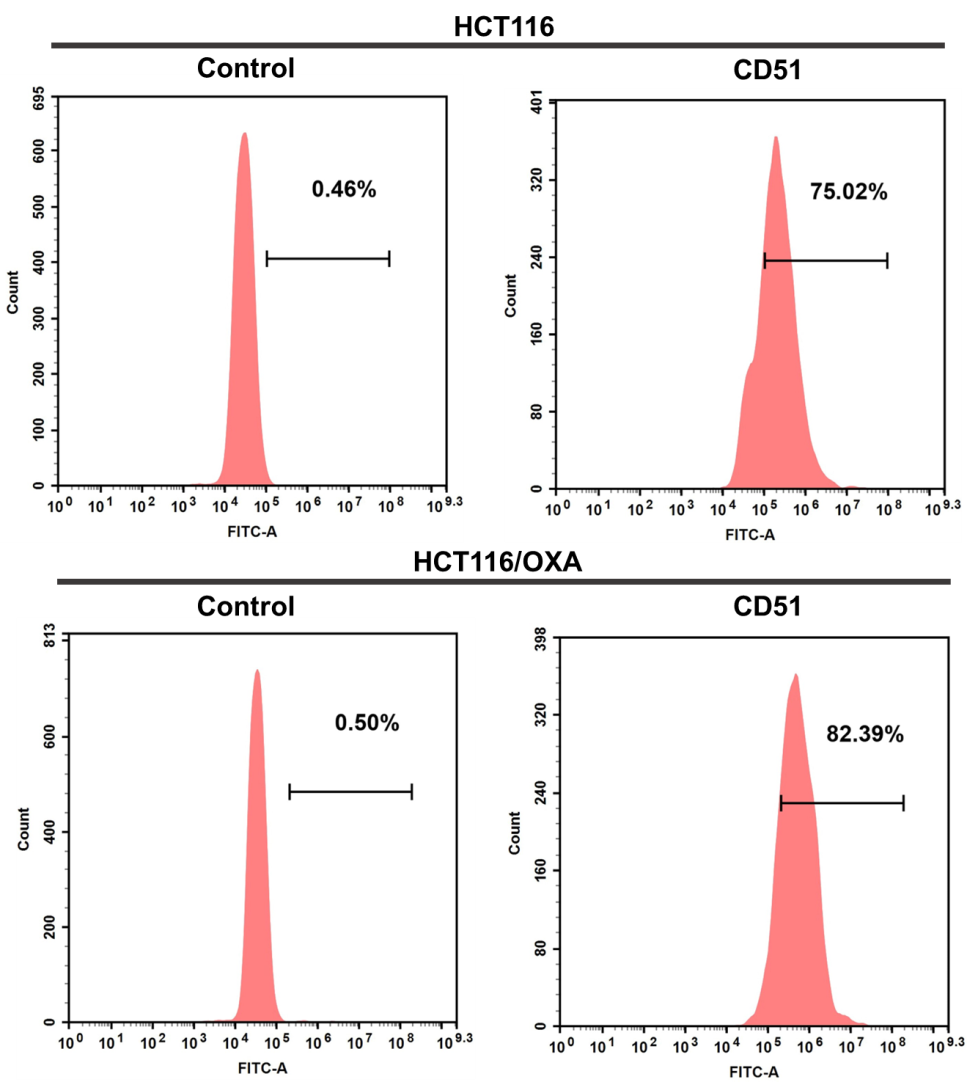


Figure S11 Flow cytometry of integrin αvβ3 content on the surface of HCT116 and HCT116/OXA cells.


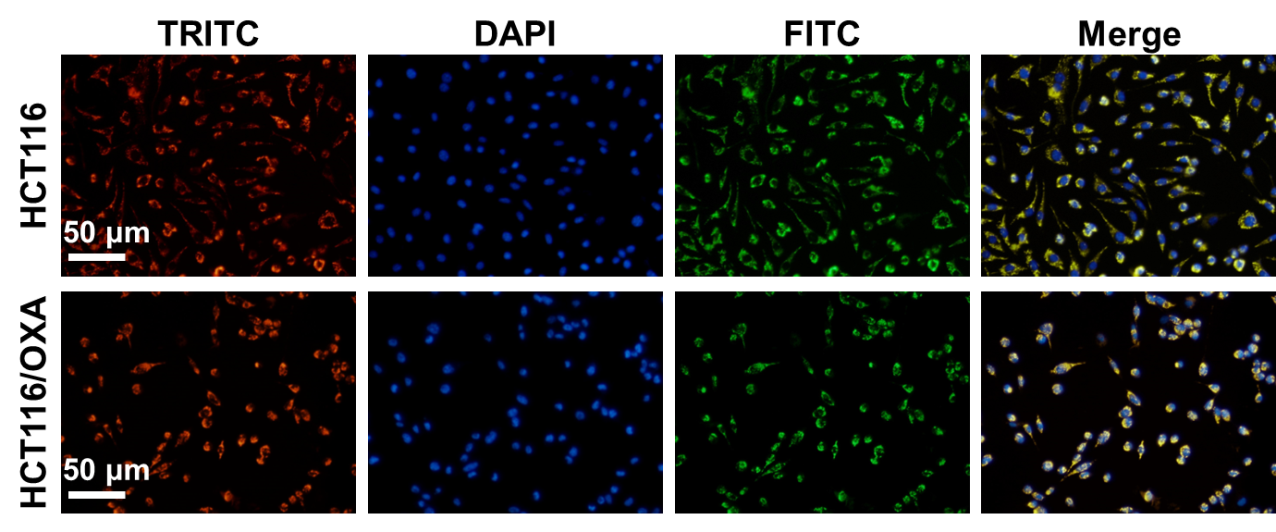


Figure S12 CLSM images of integrin αvβ3 expression in HCT116 and HCT116/OXA cells (integrin αvβ3 was labeled with FITC).


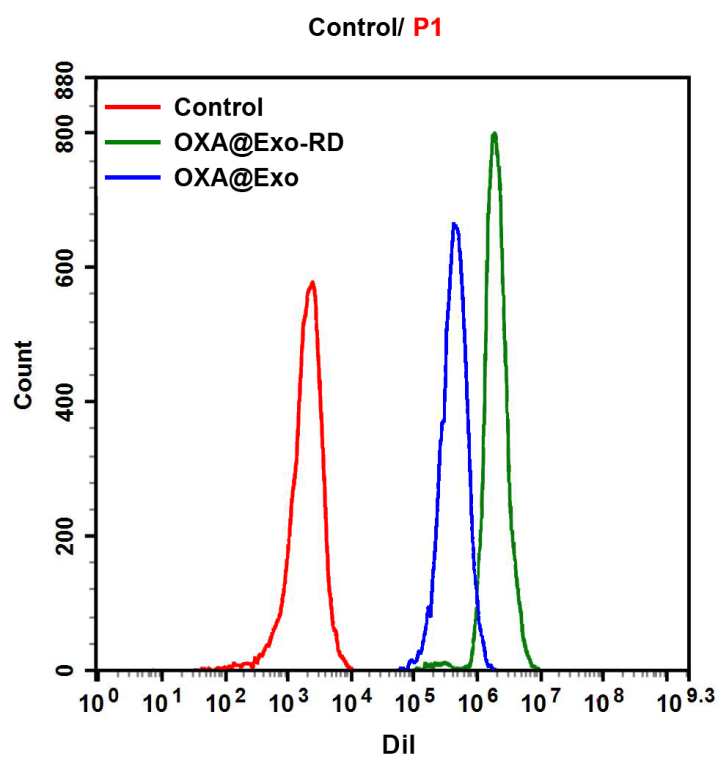


Figure S13 Cellular uptake of OXA@Exo and OXA@Exo-RD measured using flow cytometry.


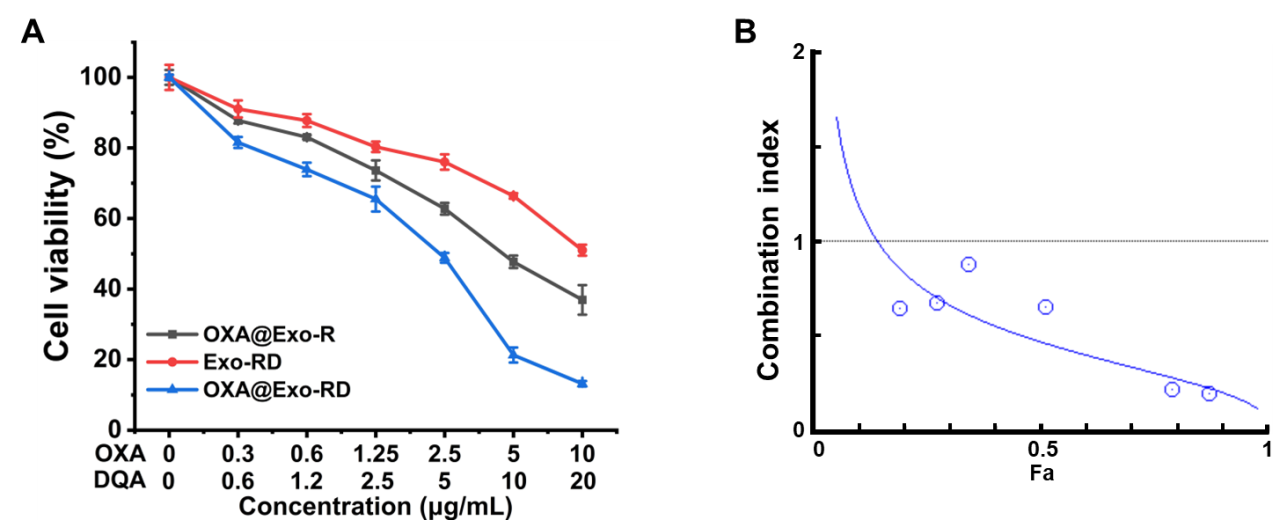


Figure S14 The synergistic antitumor activity of OXA and DQA in OXA@Exo-RD. (A) The dose-effect curve in HCT116/OXA cells after treatment with OXA@Exo-R, Exo-RD and OXA@Exo-RD for 24 h. (B) The drug cytotoxicity synergism was analyzed via CompuSyn method with a combination index (CI). CI values < 1, = 1, and > 1 indicate synergism, additive effect, and antagonism, respectively. Fa, Fraction affected.


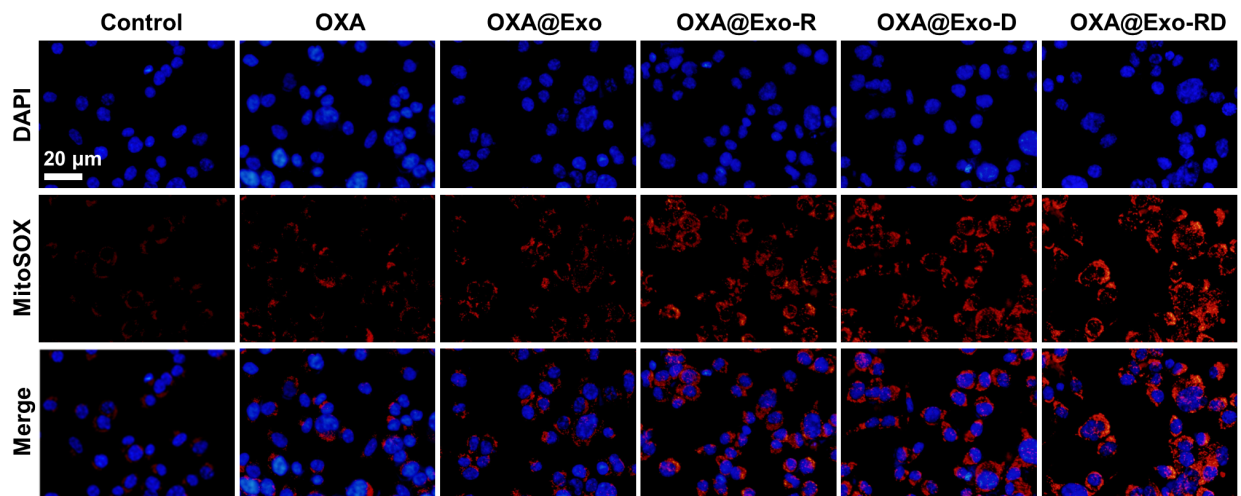


Figure S15 Representative CLSM images of mitochondrial ROS in HCT116/OXA cells after different treatments.


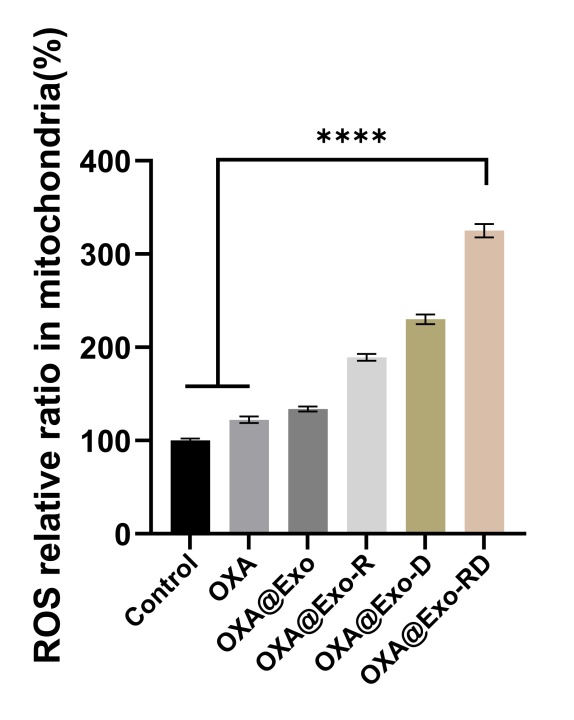


Figure S16 Mitochondrial ROS level in HCT116/OXA cells after different treatments.


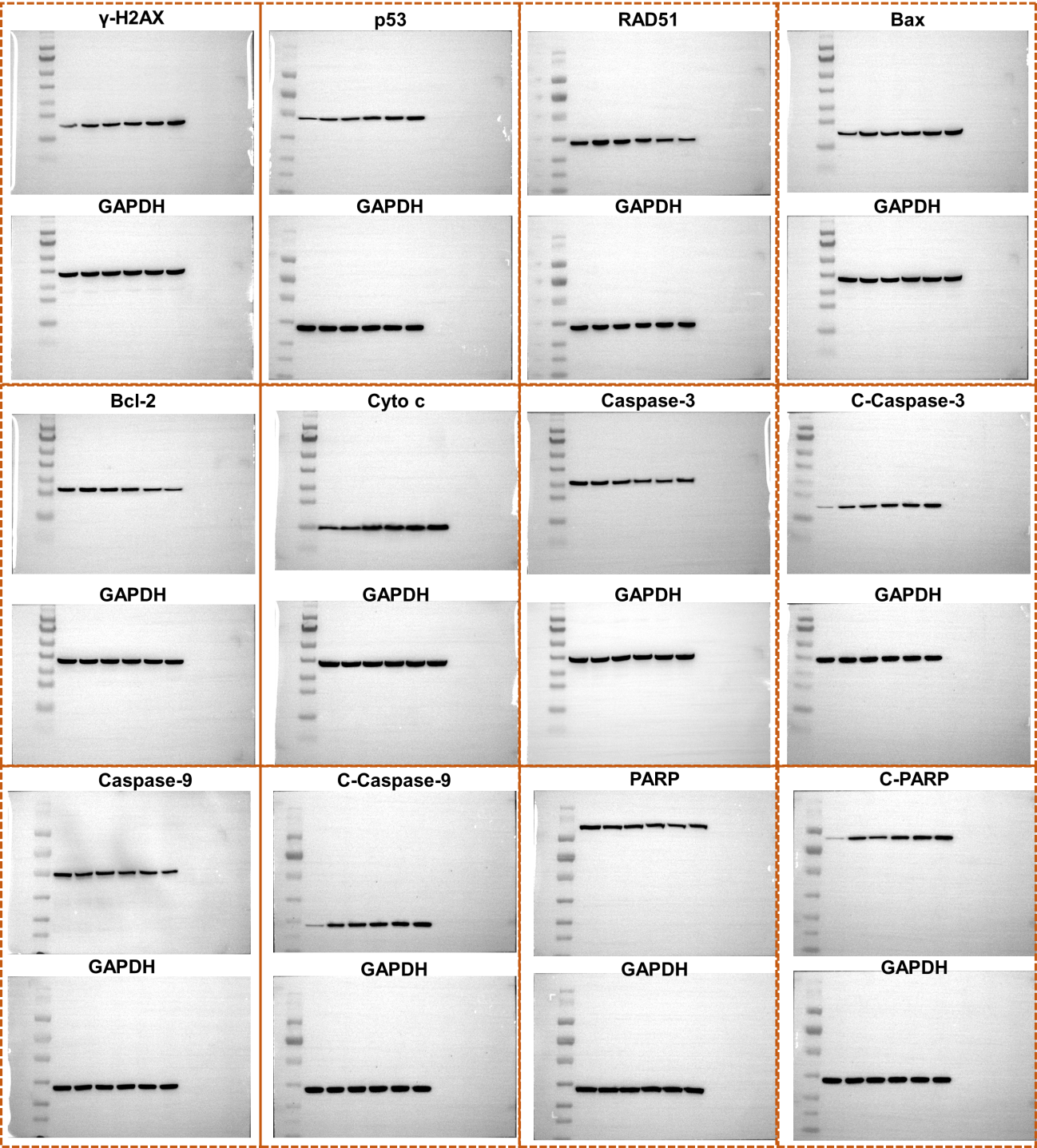


Figure S17 Western blotting raw data of apoptosis-related protein expression.


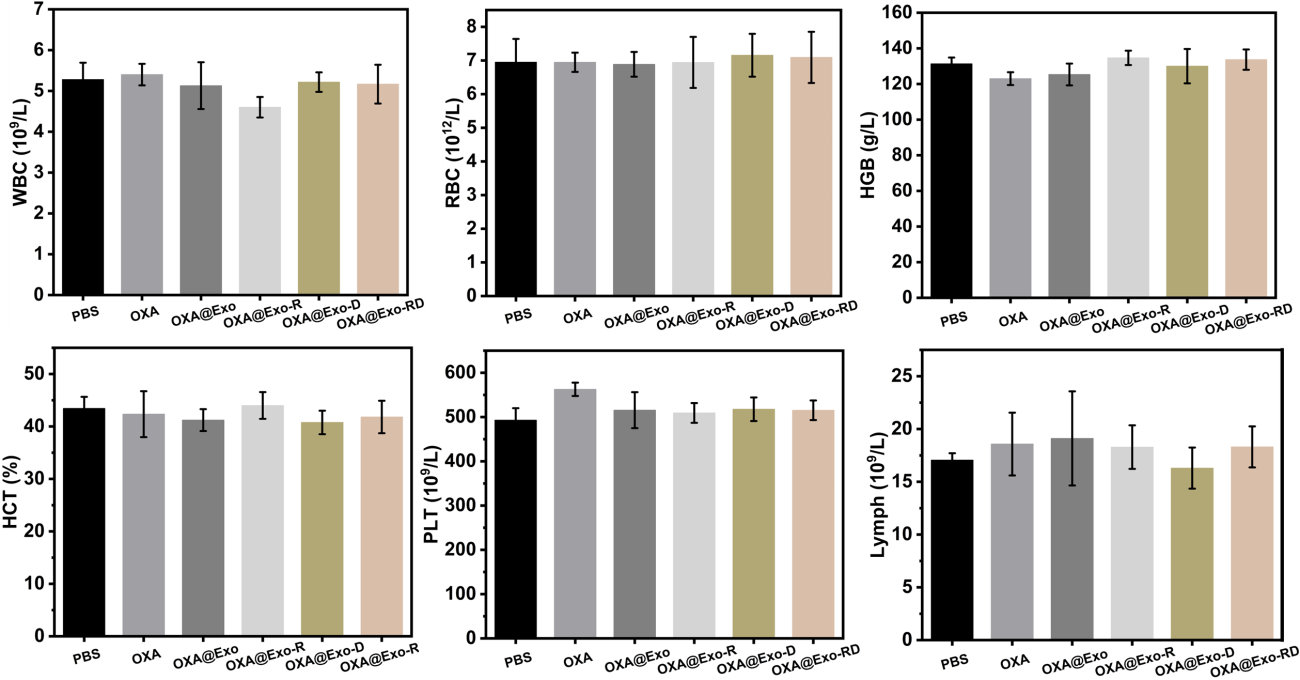


Figure S18 Blood routine analysis of different groups of mice at 21 days of treatment. (WBC = white blood cell, RBC = red blood cell, HGB = hemoglobin, HCT = hematocrit, PLT = platelets, Lymph = lymphocyte) (n = 3).


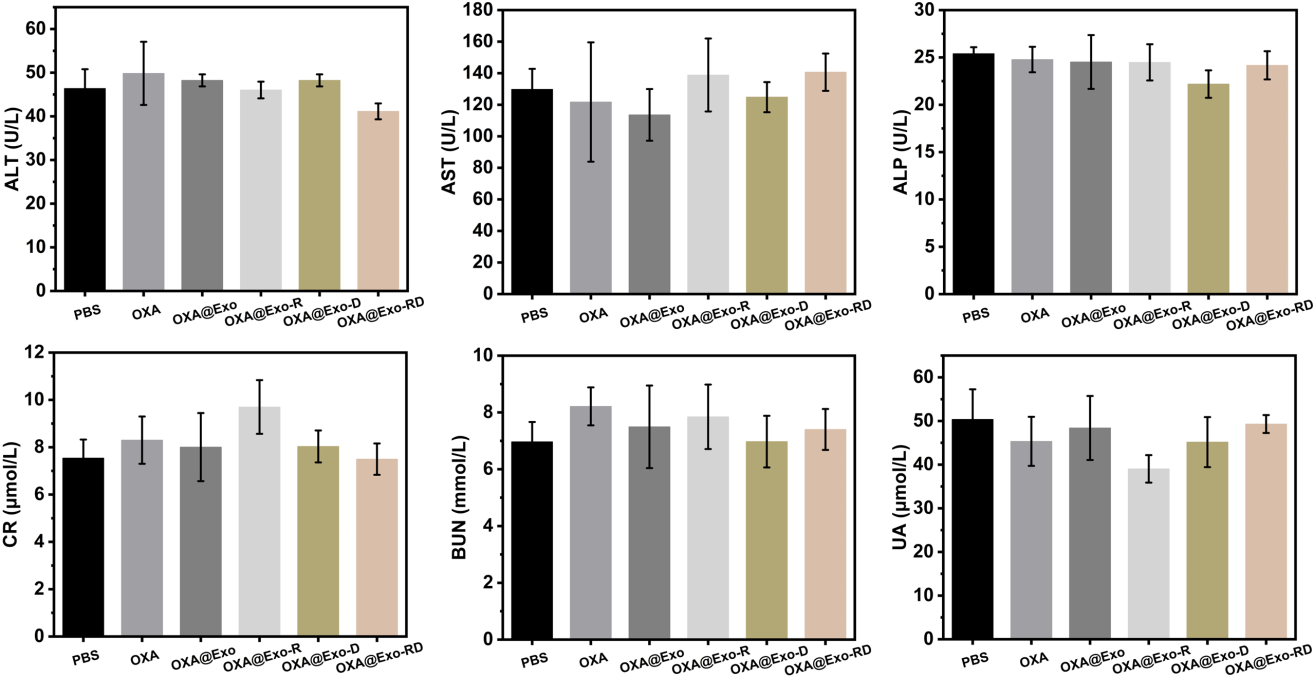


Figure S19 Blood biochemical analysis of different groups of mice at 21 days of treatment. (ALT = alanine transaminase, AST = aspartate transaminase, ALP = alkaline phosphatase, CR = creatinine, BUN = blood urea nitrogen, UA = uric acid) (n = 3).


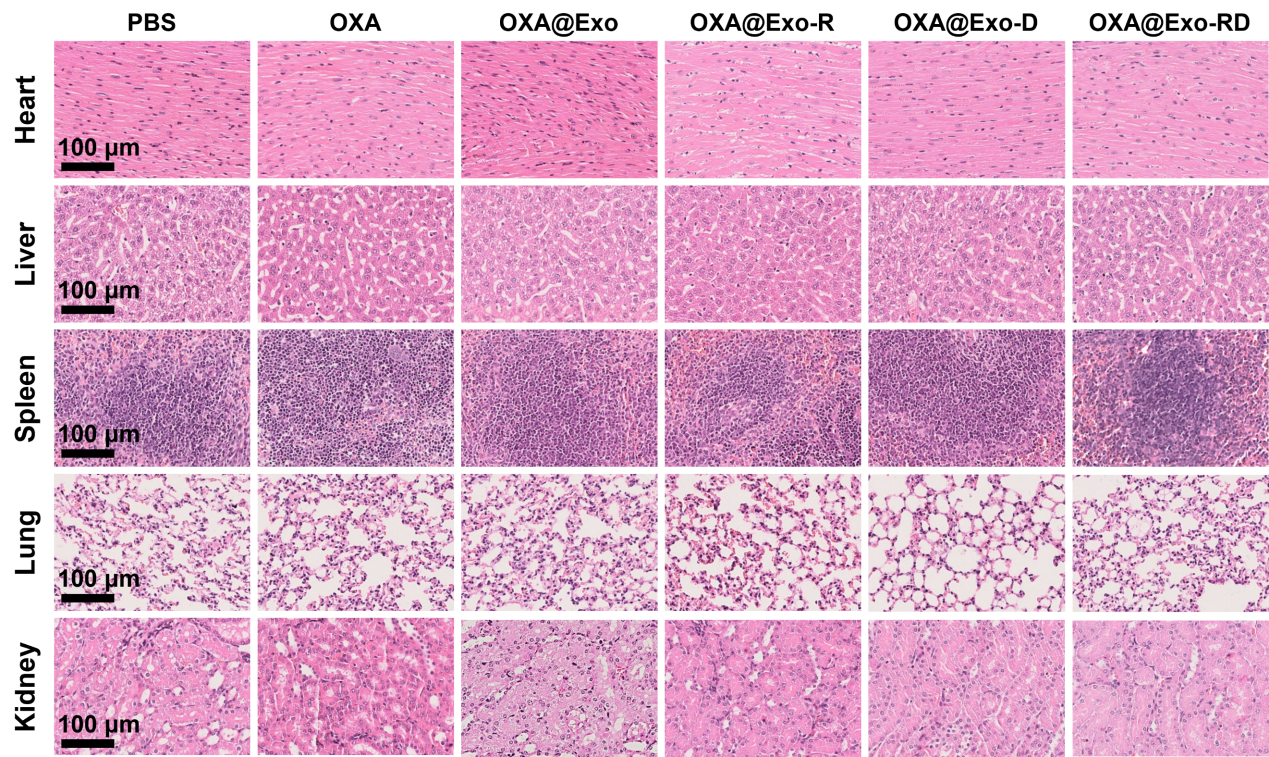


Figure S20 H&E staining images of major organs after different treatments.
